# Cytochrome c oxidase inactivation in *Physcomitrium patens* reveals that respiration coordinates plant metabolism

**DOI:** 10.1093/plcell/koaf101

**Published:** 2025-05-05

**Authors:** Antoni M Vera-Vives, Marco Mellon, Libero Gurrieri, Philipp Westhoff, Anna Segalla, Shun-ling Tan, Edoardo Bizzotto, Stefano Campanaro, Francesca Sparla, Andreas P M Weber, Alessandro Alboresi, Tomas Morosinotto

**Affiliations:** Department of Biology, University of Padova, 35131 Padova, Italy; Department of Biology, University of Padova, 35131 Padova, Italy; Department of Pharmacy and Biotechnology (FABIT), University of Bologna, 40126 Bologna, Italy; Plant Metabolism and Metabolomics Laboratory, Cluster of Excellence on Plant Science (CEPLAS), Heinrich Heine University, 40225 Düsseldorf, Germany; Department of Biology, University of Padova, 35131 Padova, Italy; Department of Biology, University of Padova, 35131 Padova, Italy; Department of Biology, University of Padova, 35131 Padova, Italy; Department of Biology, University of Padova, 35131 Padova, Italy; Department of Pharmacy and Biotechnology (FABIT), University of Bologna, 40126 Bologna, Italy; Institute of Plant Biochemistry, Cluster of Excellence on Plant Science (CEPLAS), Heinrich Heine University, 40225 Düsseldorf, Germany; Department of Biology, University of Padova, 35131 Padova, Italy; Department of Biology, University of Padova, 35131 Padova, Italy

## Abstract

Photosynthetic organisms use sunlight as an energy source but rely on respiration during the night and in nonphotosynthetic tissues. Respiration also occurs in photosynthetically active cells, where its role is still unclear due to the lack of viable mutants. Mutations abolishing cytochrome c oxidase (Complex IV) activity are generally lethal. In this study, we generated cytochrome c oxidase assembly protein 11 (*cox11*) knockout lines through vegetative propagation in the moss *Physcomitrium patens*. These mutants showed severely impaired growth, with an altered composition of the respiratory apparatus and increased electron transfer through alternative oxidase. The light phase of photosynthesis remained largely unaffected in *cox11* plants, while the efficiency of carbon fixation was reduced. Transcriptomic and metabolomic analyses showed that disrupting the cytochrome pathway had broad consequences for carbon and nitrogen metabolism. A major alteration in nitrogen assimilation was observed, with a general reduction in amino acid abundance. Partial growth rescue was achieved by externally supplying plants with amino acids but not with sugars, demonstrating that respiration in photosynthetic plant cells plays an essential role at the interface between carbon and nitrogen metabolism and a key role in providing carbon skeletons for amino acid biosynthesis.

## Teaser

Plants with impaired respiration can still perform photosynthesis but are strongly impaired in their ability to exploit the available energy for supporting metabolism, with a strong impact on growth.

IN A NUTSHELL **Background:** Plants thanks to photosynthesis convert sunlight into the chemical energy used to fuel all their cellular metabolism. Plants also rely on mitochondrial respiration during the night and in nonphotosynthetic tissues.

**Question:** The metabolic role of respiration in photosynthetically active cells is still largely unclear, also because of the lack of viable mutants.

**Findings:** The first plants depleted of Complex IV activity were generated in *Physcomitrium patens.* The mutants show a severe growth phenotype, but this is not associated with cellular energy deficiency. Photosynthetic reactions show minor alterations, because mutants are still able to consume the reducing power produced from the chloroplast. In contrast, impaired respiration affects cellular ability to use available fixed carbon for amino acid biosynthesis, demonstrating that respiration plays a key role at the interface between carbon and nitrogen metabolism.

**Next steps:** This novel genetic material will enable to investigate in further detail the impact of respiration on different plant metabolic pathways.

## Introduction

Photosynthetic organisms use light energy as their primary source of energy to synthesize the ATP and the reducing power (NADPH) needed to support their entire metabolism and especially the fixation of CO_2_ into organic molecules. This metabolic reaction is responsible for primary production, providing the chemical energy to support most life forms. Photosynthetic organisms also rely on respiration to meet energy demands at night (i.e. in the absence of light) or in tissues that are not photosynthetically active, such as roots or seeds during germination.

Oxidative phosphorylation is the main metabolic pathway responsible for generation of ATP in heterotrophic cells, and in eukaryotes, it is localized in mitochondria. This process is fed by reducing the power originating from the oxidation of organic acids and leads to the release of CO_2_ and the reduction of O_2_ to water. Oxidative phosphorylation is catalyzed by 5 large multisubunit complexes embedded in the inner mitochondrial membrane: the NADH dehydrogenase complex (Complex I [CI]), the succinate dehydrogenase complex (Complex II [CII]), the cytochrome c reductase complex (Complex III [CIII]), the cytochrome c oxidase complex (Complex IV [CIV]), and the ATP synthase (Complex V [CV]). Together, CI to CIV form the mitochondrial electron transfer chain (mETC), or respiratory chain, which catalyzes the transfer of electrons from NADH or FADH_2_ to molecular oxygen. CI, CIII, and CIV of the respiratory chain are proton translocators, and mETC activity drives the generation of an electrochemical gradient across the inner mitochondrial membrane, which is subsequently used by CV to catalyze the phosphorylation of ADP to ATP.

In addition to its role in supporting energy metabolism in heterotrophic tissues or in the dark (Millar et al. 2011), plant respiration has also been shown to be active in the light and to influence photosynthesis by acting as a sink for excess electrons coming from the chloroplasts thanks to the action of the malate-oxaloacetate valve, which allows the exchange of reducing power between organelles (Alric and Johnson 2017; Igamberdiev 2020; Igamberdiev and Bykova 2023). Mitochondrial respiration activity has been shown to increase under illumination (De Col et al. 2017) and directly affects photosynthetic electron transport (Larosa et al. 2018) and chloroplast ATPase activity (Mellon et al. 2021). In diatoms, a group of marine algae, the energetic coupling between plastids and mitochondria is essential to drive CO_2_ assimilation (Bailleul et al. 2015). A growing body of evidence suggests that respiration plays an important biological role in plants, even under illumination, and that its activity is closely related to photosynthesis.

Mitochondrial respiration is essential to supply ATP to the cytosol, even in photosynthetically active cells, as recently demonstrated in *P. patens* (Vera-Vives et al. 2024). Indeed, the inner mitochondrial membrane contains an efficient ADP/ATP exchange system (Fricaud et al. 1992; Gout et al. 2014), while chloroplasts can export ATP to the cytosol only under some conditions (Stocking and Larson 1969; Heber and Santarius 1970; Gardeström and Igamberdiev 2016). Mitochondrial respiration is an essential source of ATP for sucrose synthesis also in illuminated photosynthetic tissues (Kromer 1995; Gardeström et al. 2002; Noguchi and Yoshida 2008).

Plant mitochondria have additional roles in stress responses, contributing to tolerance to abiotic stress and the orchestration of programmed cell death (Suzuki et al. 2012; Van Aken 2021), in cellular redox regulation and the management of reactive oxygen species (Geigenberger and Fernie 2014), and in the biosynthesis of key metabolites such as amino acids, fatty acids, vitamin cofactors or tetrapyrroles (Mackenzie and McIntosh 1999; Fernie et al. 2020). Consequently, several solute transporters are present in the inner mitochondrial membrane (Fernie et al. 2020).

The synthesis of amino acids in the cytosol and plastids by glutamine synthetase/glutamine:2-oxoglutarate aminotransferase enzymes requires a supply of carbon skeletons, which are obtained through direct or indirect export of alpha-ketoglutaric acid from the mitochondrial TCA cycle (Gauthier et al. 2010). Amino acids can also be used as an electron source to fuel the mETC during carbon starvation (Szal and Podgórska 2012; Cavalcanti et al. 2017; Van Aken 2021).

Despite its biological relevance, the role of mitochondrial respiration in plants under illumination is far from understood. A major limitation in the advancement of knowledge in this area has been the lack of viable land plant mutants with depleted respiration. While there is a collection of mutants depleted in one or more respiratory complexes in the green alga *Chlamydomonas reinhardtii* (Salinas et al. 2014), complete knockout (KO) mutants in land plants are only available for CI in *Arabidopsis thaliana* (Meyer et al. 2009; Fromm et al. 2016a; Pétriacq et al. 2017), *Nicotiana sylvestris* (Gutierres et al. 1997), and the moss *Physcomitrium patens* (Mellon et al. 2021). Full KO mutants for CII to CV have never been isolated in vascular plants due to the negative effects on embryo development and seed germination.

The lethal consequences of CIV depletion have been reported several times (Steinebrunner et al. 2011; Mansilla et al. 2015; Kolli et al. 2019; Gras et al. 2020). Only plants with reduced accumulation of specific subunits of CIV have been isolated and exhibited decreased viability, developmental inhibition, sterility, and impaired seed germination (Kolli et al. 2019). These phenotypes are consistent with the energetic depletion of nonphotosynthetic tissues. A cytochrome c oxidase deficient1 (*cod1*) mutant in Arabidopsis has been reported with undetectable levels of CIV activity (Dahan et al. 2014). However, *cod1* seeds did not germinate and plants could only be obtained by rescue of in vitro embryos with very slow development that did not allow a full assessment of the metabolic impact of respiration deficiency in photosynthetic tissues (Dahan et al. 2014).

This lack of viable experimental models was here addressed using moss *P. patens* where KO mutants can be generated by homologous recombination in vegetatively propagated tissues cultivated under continuous light. Most tissues of *P. patens* are haploid, allowing direct evaluation of the final phenotype associated with any genetic modification by bypassing sexual reproduction and thus respiration-dependent developmental stages such as fertilization and seed or spore germination. Most of the life cycle of *P. patens* consists of photosynthetically active cells, and even rhizoids still contain some active chloroplasts (Sakakibara et al. 2003), making it a highly suitable model for isolating mutants with potentially altered mitochondrial functions, as recently demonstrated with the isolation of a viable CV KO mutant (Vera-Vives et al. 2024).

For generating mutants in *P. patens* depleted in CIV activity, we chose the protein cytochrome c oxidase assembly protein 11 (COX11) as a target. COX11 is an assembly factor required for the insertion of 2 copper ions into the COX1 subunit, which forms the Cu_B_ center of the CIV catalytic core (Meyer et al. 2019). COX11 is well conserved in different organisms with respiratory activity (Timón-Gómez et al. 2018; Esposti 2020). COX11 activity has been shown to be essential for CIV functionality, and its depletion led to respiratory null mutants in yeast (Banting and Glerum 2006), motivating its selection as a target to generate CIV-depleted plants. Complete KO was not achieved in Arabidopsis, while reduction of COX11 protein levels by knockdown approaches led to defective embryo development (Radin et al. 2015).

Here, we present the generation and characterization of *P. patens* plants defective in COX11, which show undetectable CIV activity and altered respiratory activity. The mutation caused a strong alteration in carbon metabolism and an inability to mobilize energy reserves. Nitrogen metabolism was also altered, likely as a result of a lack of carbon skeletons for amino acid biosynthesis, demonstrating that mitochondrial metabolic activity is essential even in photosynthetically active cells.

## Results

### Depletion of *COX11* alters the composition and activity of the respiratory apparatus in *P. patens*

In *P. patens*, COX11 is encoded by a single nuclear gene and the encoded protein sequence includes a putative mitochondrial targeting peptide (Supplementary Fig. S1). Using publicly available transcriptomic data, we found that the *COX11* gene is expressed at significant levels in all tissues, although the highest levels of transcript are detected in imbibed spores compared to other stages (Supplementary Fig. S2). Three cysteine residues known to be involved in Cu^2+^ binding and essential for COX11 activity as a copper chaperone in yeast (Carr et al. 2002) are also conserved in Pp-COX11 (Supplementary Fig. S1). All these observations are consistent with the conserved COX11 activity in *P. patens*.

**Figure 1.**
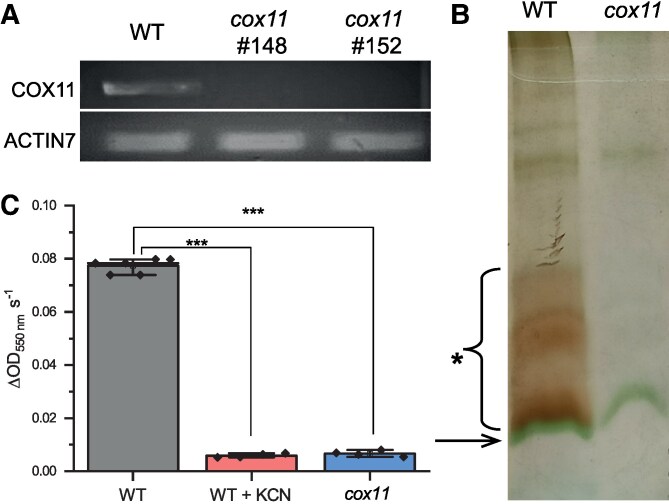
Isolation of KO mutants for the *cox11* gene in *P. patens*. **A)** RT-PCR verification of COX11 expression in the 2 independent lines #148 and #152. **B)** In-gel activity staining after separation of crude membrane extracts by BN-PAGE. The arrow marks a band that corresponds to LHCII trimers (Järvi et al. 2011), and the asterisk marks the area with CIV activity in the WT. **C)** Quantification of CIV activity in WT extracts compared to the same sample treated with specific inhibitor KCN and *cox11* (average ± Se)*. Cox11* was not distinguishable from WT treated with KCN. Statistics: 2-sample *t*-test (***P < 0.001, *n* = 4).

*P. patens cox11* mutants were generated by PEG-mediated transformation, as summarized in Supplementary Fig. S3. Stable hygromycin-resistant lines were validated by PCR to confirm insertion in the expected genomic region (Supplementary Fig. S3B). The absence of *COX11* mRNA was confirmed by reverse transcription (RT)-PCR (Fig. 1A) and RNA-sequencing (RNA-seq) analyses (reported below). To verify that loss of *COX11* led to a deficiency in CIV activity, crude membrane extracts, enriched in mitochondrial proteins, were separated by blue native PAGE (BN-PAGE). After separation, the gel activity assay for cytochrome c oxidase showed no detectable activity in *cox11* plants, which was instead clearly detected in wild-type (WT) plants (Fig. 1B; Supplementary Fig. S3). CIV activity was also quantified spectroscopically in total extracts from cytochrome c oxidation (Supplementary Fig. S3, E and F). *cox11* showed a strong reduction in activity, not distinguishable from the one of plants treated with the inhibitor KCN (Fig. 1C).

### Growth is altered in *cox11* plants

**Figure 2.**
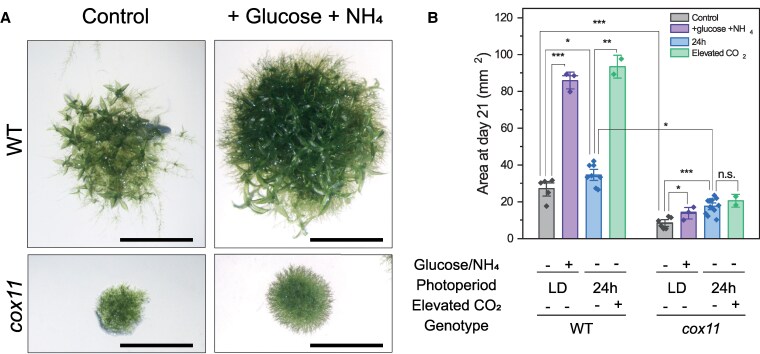
Growth phenotype of *cox11* mutants under different growth conditions. **A)** Image of a representative colony of WT and *cox11* after 21 d of growth on solid PpNH_4_ medium. The scale bar is 5 mm. **B)** Area quantification (± Se) on Day 21 of colonies grown under control conditions. LD, long day; 24 h, continuous illumination. Statistics: 2-sample *t*-test (***P < 0.001; **P < 0.01; *P < 0.05; n.s. not significant, *n* > 3). *cox11* reports merged data from 2 independent lines.

All validated *cox11* lines showed severely impaired growth and delayed developmental phases (Fig. 2). *The cox11* plants remained longer than WT in the protonema phase, and the first gametophores were observed after 21 d, while in WT, they were visible after 10 d (Fig. 2; Supplementary Fig. S4). Spore production could not be induced in *cox11* under the conditions normally effective for WT plants.

The growth phenotype of *cox11* was assessed under different conditions (Fig. 2B). The addition of glucose and ammonia to the medium had a positive effect on the *cox11* growth, as it did for WT plants, and the respective difference remained (Fig. 2B). Growth under continuous illumination caused a small but significant improvement in *cox11* growth compared to the control long-day photoperiod. However, this increase was far from sufficient to rescue the growth difference with WT, which remained very large. Finally, growth under an elevated CO_2_ atmosphere, which induces carbon fixation and removes the negative effects of photorespiration, had only a minor positive effect on *cox11*, while strongly stimulating the growth of WT plants.

### The composition and activity of the respiratory apparatus are altered in *cox11*

**Figure 3.**
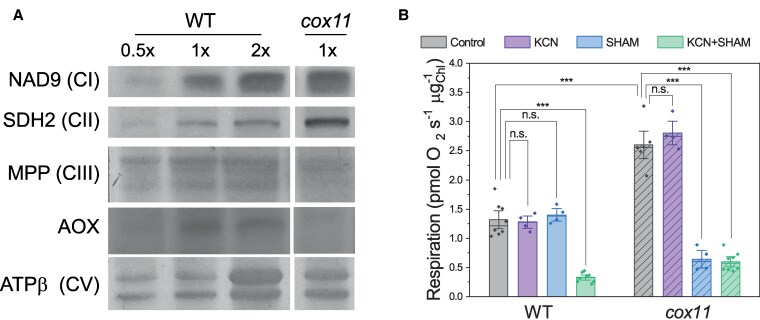
Alterations in the *cox11* respiratory machinery. **A)** Immunoblot of subunits of the different respiratory complexes. For CI, CII, CIII, and the AOX, total protein extracts were used. For CV, crude mitochondrial extracts were used. Different amounts of proteins were loaded, expressed in multiples of WT (0.5×, 1×, and 2×). 1× corresponds to 2 *μ*g of chlorophyll for NAD9, SDH2, and the AOX, 4 *μ*g of chlorophylls for MPP, and 30 *μ*g of proteins for the ß subunit. **B)** Oxygen consumption in respirometry on intact protonema (± Se). Statistics: 2-sample *t*-test (***P < 0.001; n.s. *P* > 0.05, *n* > 4).

The effect of *COX11* depletion on respiratory apparatus was verified by immunoblotting, using antibodies against some of core proteins of the respiratory complexes (Fig. 3A). Even though the absolute quantification may be affected by the slower growth of the mutant, this analysis showed relatively increased levels of the CI core subunit NAD9, the CII core subunit SDH2, and the ß subunit of CV. Interestingly, the levels of the CIII MPP subunit were lower in *cox11* than in WT plants. Rather surprisingly, the protein levels of alternative oxidase (AOX), which allows electrons to bypass the cytochrome pathway, were also slightly lower in *cox11* than in WT plants.

Respiratory activity was assessed by measuring O_2_ consumption in the dark in intact protonema tissues (Fig. 3B). Interestingly, O_2_ consumption in the dark, normalized to the Chl content, was increased in *cox11* lines, reaching approximately twice the activity of WT plants. This was independent of normalization to the amount of protein or chlorophylls, since the protein/chlorophyll ratio was unchanged between WT and *cox11*, with values of 90.7 ± 4.0 (WT) and 90.8 ± 0.6 (*cox11*) mg_prot_/mg_Chl_.

Even though the difference in growth rate can impact the activity, these data clearly suggest that *cox11* plants retained significant capacity. In order to understand its origin, O_2_ consumption measurements were repeated after the addition of chemicals that inhibit cyanide-sensitive or the cyanide-insensitive pathways, i.e. potassium cyanide (KCN) or salicylhydroxamic acid (SHAM), respectively. In WT, the effects of KCN or SHAM on dark respiration were not significant when applied alone (Fig. 3B), while when applied together, they abolished virtually eliminated all the dark respiration activity (Fig. 3B). This suggests that when the cytochrome pathway is chemically blocked, the excess of electrons can be readily redirected through the AOX, which thus must have a very high capacity in *P. patens* WT but is normally only potential and not translated in activity. To confirm that this effect is not due to chemical specificity, we repeated the experiment using 2 alternative chemicals, antimycin A and n-propylgallate, and reached the same conclusions (Supplementary Fig. S5A). In *cox11*, as in WT, KCN had no effect on O_2_ consumption (Fig. 3B). However, the addition of SHAM alone was able to completely inhibit O_2_ consumption (Fig. 3B). This strongly suggests that the higher rate of respiration in *cox11* is largely due to the increased activity of the cyanide-insensitive AOX-mediated pathway. This pathway already had a large capacity in WT plants where it was underexploited. The same effect was observed when the alternative AOX inhibitor n-propylgallate was used (Supplementary Fig. S5, B and C).

These results confirm that *cox11* plants lack a detectable CIV activity and that this loss completely compromises the cyanide-sensitive electron transport pathway. At the same time, the activity of the cyanide-insensitive pathway, present but underexploited in WT plants, increased in *cox11*, and it is responsible for maintaining consumption of O_2_ during dark respiration.

### Carbon fixation rather than light conversion is altered in *cox11*

**Figure 4.**
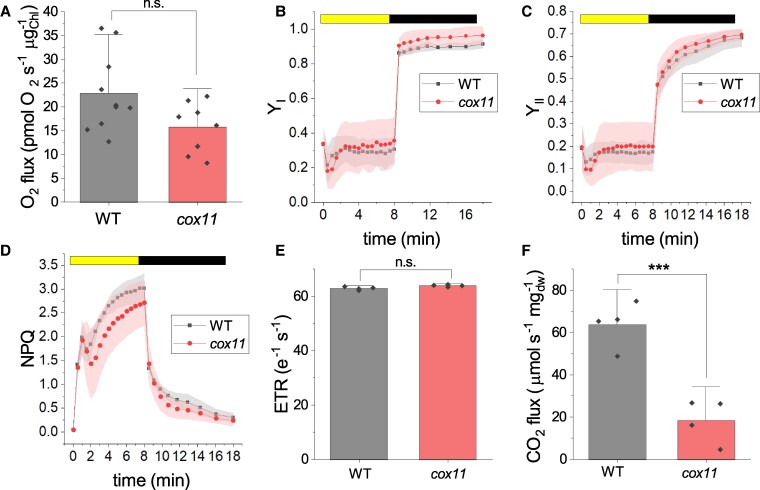
Evaluation of photosynthetic properties in *cox11*. **A)** Gross evolution of O_2_ under saturating illumination (*n* > 6). **B** to **D)** The yield of PSI (YI, **B**), PSII (YII, **C**), and nonphotochemical quenching (NPQ, **D**) was measured with Dual PAM 100 in plants exposed to 330 *μ*mol photons m^−2^ s^−1^ of actinic light intensity. Bright and dark bars at top indicate when actinic light was turned on and off, respectively. WT and *cox11* are shown, respectively, with black squares and red circles. Data are shown as average ± Sd (*n* > 4). No statistically significant differences were identified from the WT plants. **E)** ETR of dark-acclimated plants grown under dim light, calculated from the ECS (electrochromic shift signal) after exposition to saturating light (300 *µ*mol photons m^−2^ s^−1^) for 3 to 5 min. Activity was normalized to the total photosystem content (PSI + PSII). The standard deviation is also reported (*n* > 6). **F)** CO_2_ assimilation under a control light of 50 *µ*mol photons m^−2^ s^−1^ (*n* = 4). The error bars in **A)**, **E)**, and **F)** represent 1.5 times the Sd. Statistics: 2-sample *t*-test (n.s.*P* > 0.05; ***P < 0.001).

Mitochondria are active in photosynthetic tissues during the day and that they act synergically with chloroplasts to ensure optimal photosynthetic performance (Tcherkez et al. 2017; Shameer et al. 2019; Vanlerberghe et al. 2020). Therefore, we evaluated whether alterations in respiratory efficiency in *cox11* would have any effect on their photosynthetic performance. We quantified the abundance of different components of the photosynthetic machinery by immunoblotting and found no major differences (Supplementary Fig. S6). We then quantified the photosynthetic activity of the protonema pieces by measuring the rate of O_2_ evolution under saturating illumination, which induces maximal photosynthesis. In *cox11*, the net photosynthetic activity was reduced (Supplementary Fig. S7), but this was attributable to increased respiration, as gross photosynthesis was not different from WT (Fig. 4A), suggesting the performance of the photosynthetic light reactions is maintained in the mutant.

Photosynthetic activity was further assessed by chlorophyll fluorometry (Fig. 4, B to D). The efficiencies of both PSI and PSII upon exposure to subsaturating light, quantified from Y_I_ and Y_II_, respectively, were indistinguishable between WT and *cox11* (Fig. 4, B and C). The induction of nonphotochemical quenching (NPQ), a photoprotective mechanism activated by a decreasing pH in the thylakoid lumen, was also unchanged (Fig. 4D). Finally, the photosynthetic electron transport rate (ETR), quantified at steady state, was also indistinguishable between the WT and *cox11* plants (Fig. 4E).

The efficiency of the metabolic phase of photosynthesis was evaluated by quantifying the protonema CO_2_ fixation rate under a control light of 50 *µ*mol photons m^−2^ s^−1^ (Fig. 4F), which was significantly lower in *cox11* compared to WT.

Overall, these data indicate that the light phase of photosynthesis was not significantly altered in *cox11* mutants. On the contrary, net carbon fixation was affected under moderate illumination, suggesting an altered ability to use harvested energy.

### The *cox11* transcriptome shows alterations in metabolism that are dependent on the photoperiod

To globally assess the impact of the mutation on metabolism, WT and *cox11* were used for transcriptome analysis by RNA-seq, an analysis that was also unable to detect *cox11* transcript in mutants. Since it was expected that the impact of altered mitochondrial activity could change during the day, depending on whether photosynthesis is active or not, plant material was harvested at different zeitgeber times (ZTs), that is, at the end of the night (ZT0), at the beginning (ZT2), or at the middle (ZT6) of the day (Supplementary Fig. S8).

**Figure 5.**
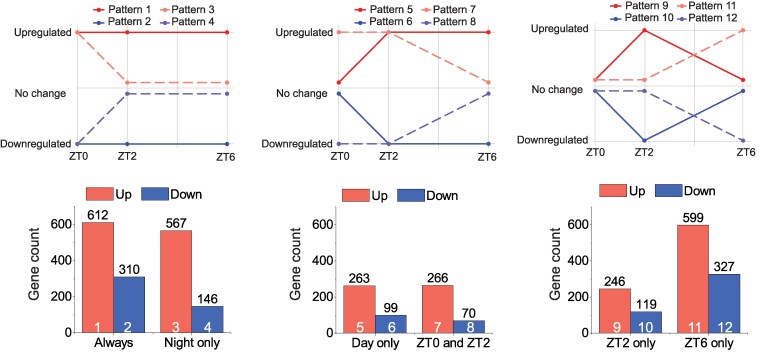
Overview of diel regulation of the DEGs identified in *cox11*. We define 12 different patterns and manually classify the genes accordingly (top). The number of genes included in each group is shown in the column charts (bottom). The numbers on top of the bars show the number of genes included. The numbers in the bars identify the corresponding pattern. The lists of genes following each of the patterns are supplied in Supplementary Data Set S1C.

After comparing the lists of differentially expressed genes (DEGs) at each of the 3 time points tested, we classified the DEGs according to the time of day they were altered, defining 12 different expression patterns (Fig. 5; Supplementary Data Set S1). In all cases, the number of genes upregulated in *cox11* was larger than the number of downregulated genes. We performed pathway enrichment analysis on DEGs at each of the 3 time conditions (Supplementary Fig. S8C) and compared pathways that were exclusive or shared between conditions (Supplementary Fig. S9 and Data Set S2).

There were 612 genes significantly upregulated under all conditions (Pattern 1), although we did not identify any specific pathway that was always significantly upregulated. On the other hand, we identified 310 genes and 46 pathways that were always downregulated in *cox11* compared to WT (Pattern 2; Supplementary Fig. S9A). Several of these genes are involved in cell wall biogenesis and remodeling or in the metabolism of structural polysaccharides, suggesting alterations in cell wall architecture (Le Gall et al. 2015). The expression of various transporters was also reduced in *cox11*, including transporters of amino acids, inositol, ammonium, phosphate, or zinc, as well as several aquaporins. This could reflect a reprogramming of the cell metabolic machinery to cope with a strongly reduced growth, which necessarily reduces cell wall synthesis and elongation, as well as nutrient demand (Boursiac et al. 2008; Vandeleur et al. 2014).

Seven hundred thirteen genes showed differential expression (146 down and 567 up) only at night, but unchanged levels during the day. Upregulated pathways at night (Pattern 3; Supplementary Fig. S9B) included a group of transaminases involved in amino acids, suggesting that nitrogen metabolism might be altered in *cox11*, particularly at night. Following Pattern 3, we also detected several genes associated with stress responses that included many different types of heat shock proteins (Hsp), either from the small (Hsp17.4, Hsp17.6, and Hsp20) or large (Hsp90) families (Waters and Vierling 2020), as well as proteins of the BAG family, which are likely chaperone regulators and might assist heat shock proteins in their chaperone role (Kabbage and Dickman 2008).

Induction of heat shock proteins could be part of a more general response to accumulation of unfolded proteins, which would trigger the unfolded protein response in the endoplasmic reticulum (UPR^ER^). However, the expression levels of some genes previously defined as markers of UPR^ER^ in *P. patens* (Lloyd et al. 2018) were not significantly higher in *cox11* (Supplementary Table S1), therefore not supporting a general induction of UPR^ER^ in *cox11*. We also checked the expression levels of genes encoding antioxidant enzymes, but none was significantly upregulated in *cox11* plants (Supplementary Table S2), suggesting that plants were not suffering from general oxidative stress. The only exception was a superoxide dismutase, PpFSD2, predicted to be aimed at the chloroplast (Higashi et al. 2013; Supplementary Table S2), which was always induced in *cox11* plants. Therefore, the expression pattern of the different heat shock proteins suggests that a stress response is induced during the night, but is then alleviated during day, likely by exposure to light, which apparently can relieve the stress. The induction of heat shock proteins is likely not due to oxidative stress, because antioxidants did not get induced.

The genes repressed at night (Pattern 4; Supplementary Fig. S9B) included one of the 3 annotated genes that encode nitrate reductase isoforms in *P. patens*, a key enzyme for nitrogen assimilation (Chamizo-Ampudia et al. 2017) whose activity can be regulated during abiotic stress (Fu et al. 2018). In addition, there was a group of 35 transcription factors (TFs) of different families, including apetala 2 (AP2), MYB, MYC, ABA inducible, and GRAS. Some of these TFs also retained their regulation during the first hours of day (Pattern 8; Supplementary Fig. S9D), but all recovered normal values at ZT6. AP2, with 149 members in *P. patens*, has been linked to defense and stress responses, growth, and development (Aoyama et al. 2012; Ishikawa et al. 2019; Zhang et al. 2020). In turn, MYB factors have been linked to protonema growth and the transition from chloronema to caulonema (Pu et al. 2020) and GRAS factors to the development of gametophores and spores (Beheshti et al. 2021). In general, altered levels of these TFs are likely correlated with the delay in the development program observed in *cox11*.

We found sets whose expression was normal at night but changed during the day. Most of those upregulated only at the beginning of the day (Pattern 9; Supplementary Fig. S9E) encoded chloroplast proteins, including starch synthases, required for carbon storage (Pfister and Zeeman 2016); arogenate dehydratases, required for biosynthesis of the amino acid phenylalanine (Maeda and Dudareva 2012); and ATP/ADP transporters, which could be linked to the intercompartment trafficking of energy equivalents. On the other hand, transcripts found at higher levels throughout the day (Pattern 5; Supplementary Fig. S9C) included 6 genes related to starch biosynthesis, starch synthases, or ADP-glucose synthases, which catalyze the commit step of starch biosynthesis (Stitt and Zeeman 2012; Pfister and Zeeman 2016), and several genes involved in ribosome biogenesis, transcription, and translation suggesting that gene expression and protein synthesis rates increase in *cox11*, particularly during day.

Some genes were instead repressed only at the beginning of the day (Pattern 10; Supplementary Fig. S9E), including 2 putative urea transporters (Pp3c14_7160 and Pp3c1_21890), and enzymes required for amino acid or glucose metabolism, including many glycolysis enzymes. This observation further strengthens the previously proposed hypothesis that *cox11* plants have altered central metabolism in night and at the night-to-day transition, affecting both N and C metabolism.

Genes encoding components of the respiratory complexes from *P. patens* or proteins that are involved in their assembly were identified based on similarity with sequences from *Bos taurus*, *Saccharomyces cerevisiae*, *A. thaliana*, and *C. reinhardtii* (Cardol et al. 2005; Terasawa et al. 2007; Subrahmanian et al. 2016; Kaye et al. 2019; Zancani et al. 2020). The impact of CIV inactivation on their expression levels was specifically analyzed for all detectable transcripts, as reported in Supplementary Data Set S3. This analysis showed a clear trend where a large set of genes encoding for CI (26 out of the 79 detected) were overexpressed but in most cases only after 6 h of illumination, while their expression was not altered in ZT0 and ZT2. The same trend was observed for genes encoding for Cyt c, and CV (11 out 26) that also showed several overexpressed genes in the mutant but mostly at ZT6 only. Interestingly, genes encoding for CIV also showed a similar trend with 16 out of 33 detected genes overexpressed in ZT6 except for the *cox11* gene that was not detected in the mutant, consistent with its deletion. On the contrary, only a few genes from CII and CIII showed overexpression and only in 1 time point.

Overall, these data suggest that in the *cox11* KO mutant, there is a signal that depends on the presence of light and that similarly affects genes involved in respiration, in particular the ones encoding for CI, CIV, and CV.

### Metabolomics and integrated pathway analysis show extensive alterations in carbon and nitrogen metabolism in *cox11*

**Figure 6.**
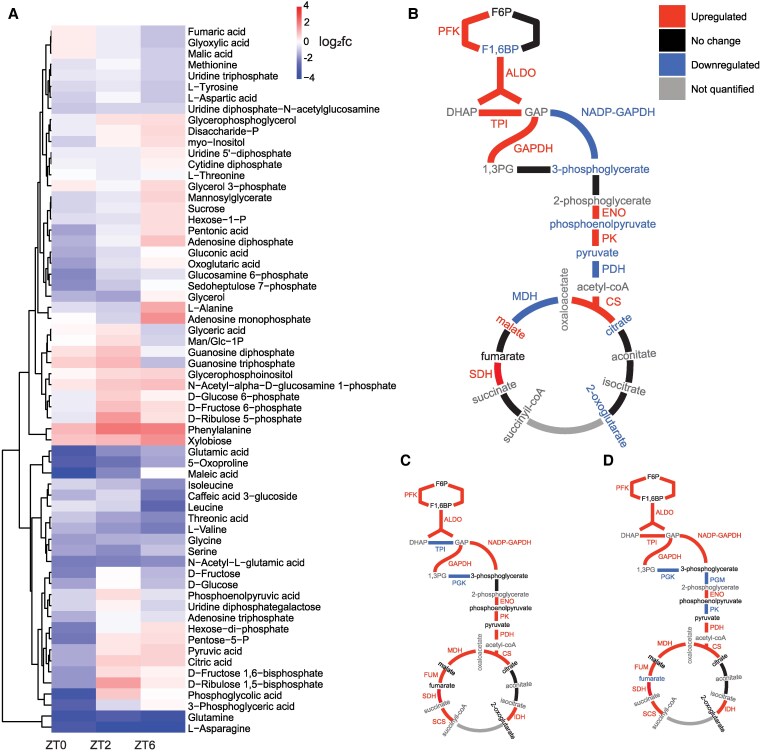
Impact of *cox11* depletion on metabolome. **A)** Heatmap showing the hierarchical clustering of compounds, fc, fold change. **B** to **D)** Representation of the metabolic pathway of glycolysis, pyruvate dehydrogenase, and TCA cycle integrating transcriptomic and metabolomic data for *cox11* at ZT0 **B)**, ZT2 **C)**, and ZT6 **D)**. Enzyme names: PFK, phosphofructokinase; ALDO, aldolase; TPI, triose phosphate isomerase; NADP-GAPDH, NADP-specific glyceraldehyde dehydrogenase; GAPDH, NADH-dependent glyceraldehyde dehydrogenase; PGK, phosphoglycerate kinase; PGM, phosphoglycerate mutase; ENO, enolase; PK, pyruvate kinase; PDH, pyruvate dehydrogenase; CS, citrate synthase; IDH, isocitrate dehydrogenase; SCS, succinyl-CoA synthetase; SDH, succinate dehydrogenase; FUM, fumarase; MDH, malate dehydrogenase.

To further investigate the alterations in *cox11* shown by the transcriptomic data, we performed untargeted metabolomics on the same samples used for transcriptome analyses. We uniquely identified a total of 65 compounds, 23 by GC-MS and 42 by IC-MS, including most primary amino acids except for arginine, cysteine, histidine, lysine, proline, and tryptophan; several monosaccharides; intermediates of glycolysis and TCA cycle; CBB cycle intermediates; and nucleotides, among other compounds (Fig. 6A; Supplementary Data Set S4). Pairwise comparisons of samples at each ZT identified 34 metabolites at significantly higher or lower levels in one or more conditions.

As we observed from the transcriptome, the largest number of differences in the *cox11* metabolome emerged when comparing the samples at night and early in the morning. In general, the metabolomic data are consistent with the transcriptome in showing important changes in carbon metabolism (Fig. 6B). Seven out of the 10 intermediates of the glycolytic pathway were identified as altered in our experiments and accumulated at lower levels in *cox11* at ZT0. Three of the 4 intermediates of the TCA cycle detected by our metabolomics approach (citrate, 2-ketoglutarate, fumarate, and malate) were less abundant while only fumarate content was not altered. Globally, metabolomic data strongly suggest that carbon catabolism was blocked or slowed in *cox11* at night (Fig. 6B). Interestingly, this alteration was no longer observed during the day, when intermediates returned to their normal values despite more general transcriptomic activation (Fig. 6, C and D). In particular, at ZT2, we observed the accumulation of both ribulose-5-phosphate and ribulose-1,5-bisphosphate, the carbon skeleton required for Rubisco to fix CO_2_ and its direct precursor; this is consistent with the reduced rate of carbon fixation that previously reported (Fig. 4F).

Nitrogen metabolism was also clearly altered in *cox11* plants. Phenylalanine was the only proteinogenic amino acid identified at higher levels in *cox11* at the beginning of the day, while the levels of the other detected amino acids were all reduced in *cox11* at ZT0 only (glycine), at ZT0 and ZT2 (asparagine, glutamate, serine, and valine) or always (glutamine) (Fig. 6A; Supplementary Data Set S4). Lower levels of glutamate, glutamine and asparagine are particularly significant, as they are among the main metabolites involved in nitrogen assimilation from inorganic nitrogen sources in plants (Gaufichon et al. 2010; Liao et al. 2022). The glutamate/glucose and aspartate/asparagine ratios were also altered (Supplementary Fig. S10), further suggesting a reduction in nitrogen assimilation efficiency. Arginine, with its high nitrogen-to-carbon ratio, is also used for nitrogen storage and mobilization in plants (Winter et al. 2015; Yoneyama and Suzuki 2020). We could not detect arginine in our metabolomic analysis, but we found the arginine biosynthetic machinery to be transcriptionally repressed. Furthermore, N-acetyl-L-glutamate, a substrate for glutamine-regulated arginine biosynthesis (Chellamuthu et al. 2014), was always found at lower levels in *cox11*.

All these observations are consistent with *cox11* cells modulating their metabolism toward a starvation situation by blocking the energetically costly process of nitrogen assimilation at night. Therefore, nitrogen assimilation and mobilization are therefore altered in *cox11*, especially at night, and can affect a plethora of metabolic and cellular processes.

### Starch mobilization is impaired, and energy availability is unchanged in *cox11*

**Figure 7.**
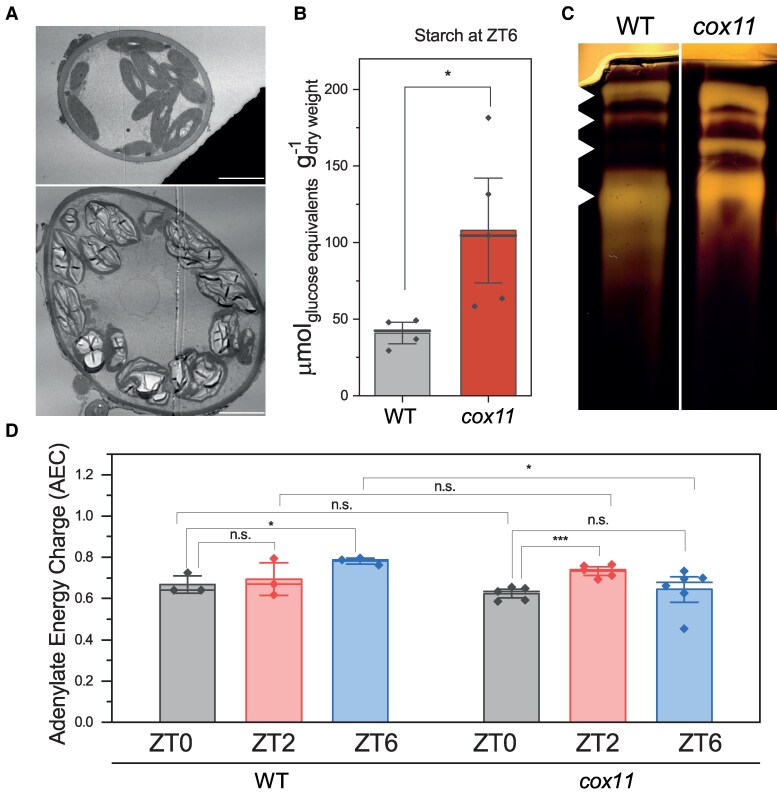
Starch accumulation and energy availability in *cox11*. **A)** Representative micrographs of WT and *cox11* cells showing differences in the amount and dimension of starch granules inside chloroplasts. Plant samples were fixed after 16 h of darkness. The scale bar is 5 *µ*m. **B)** Quantification of starch in total extracts harvested at ZT6 (± Se). Statistics: 2-sample *t*-test (*P < 0.05). **C)** In-gel activity of starch-degrading enzymes. The heads show the 4 main identified bands of activity. It is not possible to identify the enzymes responsible for each amylolytic activity because of the lack of literature data on amylases of *P. patens*. **D)** Relative AEC of WT and *cox11* (± Se). Statistics: 2-sample *t*-test (***P < 0.001; *P < 0.05; n.s. *P* > 0.05, *n* > 3).

By observing micrographs of moss samples by transmission electron microscopy, we found that *cox11* is generally larger and accumulates large starch granules in their chloroplasts. Even after 16 h of incubation in the dark, while starch granules were absent or relatively small in WT plants, they were large and occupied most of the chloroplast area in *cox11* (Fig. 7A). The quantification of the starch content in ZT6 indeed confirmed that *cox11* had more than twice the amount of starch compared to the WT (Fig. 7B). To determine whether the starch degradation capacity of *cox11* was altered, we separated total *cox11* extracts under native conditions in gels containing solubilized potato starch and estimated total and maximum amylolytic activity by in-gel activity. This test clearly shows that amylolytic activity was higher in *cox11* than in WT plants (Fig. 7C). In addition, at least 1 additional band was detected in *cox11*. These data suggest that the hydrolytic activity in *cox11* was not reduced but, on the contrary, it was increased compared to WT. Therefore, starch accumulation cannot be explained by a reduced capacity for amylolytic activity and most likely involves an increased rate of synthesis and/or reduced degradation in vivo due to the blockade of downstream metabolic processes such as glycolysis.

The previously reported deficiency of glycolysis intermediates, together with the accumulation of starch, suggests a deficient mobilization of carbon stores during the night in *cox11*. A way to measure the amount of energy available for the cell is to quantify the adenylate energy charge (AEC), based on the levels of ATP, ADP, and AMP (Atkinson 1968). We used adenylate levels from untargeted metabolomics to calculate the relative AEC values for both WT and *cox11* plants (Fig. 7D; see the Material and methods section for calculation details). We found that AEC values in *P. patens* WT plants ranged between 0.6 and 0.8, a range consistent with previous experiments in other plants (Hampp et al. 1982; Lange et al. 2008). In WT, AEC increased throughout the day, with the difference becoming significant in ZT6. In *cox11*, the value at ZT0 was not significantly different from that of WT; then, we observed an increase at ZT2 similar to that of WT, but then the AEC dropped almost to ZT0 values as the day progressed (Fig. 7D). Even if some alterations were indeed present, these data suggest that *cox11* plants do not suffer from major energy starvation.

### The external supply of amino acids partially rescues the growth phenotype of *cox11* plants

Given the general alteration in amino acid levels and metabolism, we investigated whether external application of amino acids could rescue the growth phenotype of *cox11* by providing metabolic intermediates that could restore one or more essential metabolic pathways that were impaired in *cox11* under control conditions. Therefore, we compared the growth of *cox11* plants grown under control conditions, i.e. where the sole nitrogen source was inorganic nitrate, or in enriched media where the nitrogen source was both inorganic nitrate and organic nitrogen in the form of 1 type of amino acid.

In WT plants, all amino acids except for glutamate had a significant positive or negative effect on growth that could be observed during the first 2 to 4 wk of culture in most cases (Supplementary Fig. S11A). All amino acids that reduced the growth rate of WT also had a negative effect on cox11, except phenylalanine, which did not cause a growth reduction in cox11. For some of these (histidine, isoleucine, lysine, and proline), negative effects on *cox11* were however no longer observed after 7 or more wk of culture.

**Figure 8.**
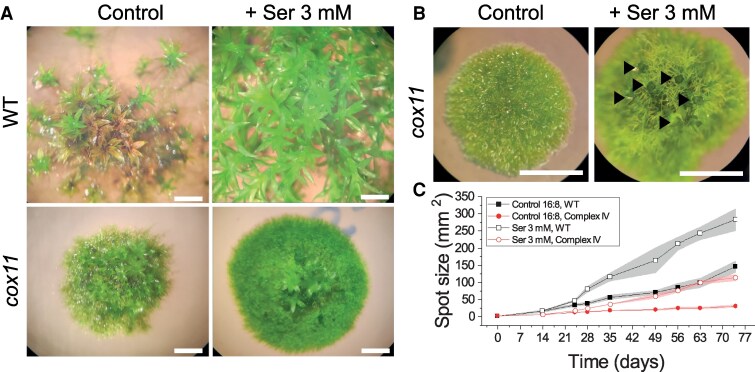
Effect of external serine supply on growth. **A)** Images of moss colonies after 42 d of growth in control medium or medium containing 3 mm serine. Scale bars are 2 mm. **B)** Detailed view of 21-d-old *cox11* plants grown in medium with or without the addition of 3 mm serine. Black arrows mark the development of gametophores. Scale bars are 2 mm. **C)** Growth curve of *cox11* supplemented with 3 mm serine (± Se), *n* > 3.

Amino acids that had a positive effect on WT (alanine, arginine, asparagine, aspartic acid, glutamine, glycine, and serine) were also beneficial for *cox11*, and in some cases, the growth improvement of *cox11* was enough to rescue the growth impairment associated with the mutation (Supplementary Fig. S11B). Serine is reported as an example of an amino acid with a strong beneficial effect (Fig. 8), and its addition not only increased plant growth but also induced an earlier formation of phyllids and gametophore-like structures on *cox11* (Fig. 8B), suggesting a rescue of the developmental program as well. The same was also observed for glycine, which is not surprising considering that serine and glycine are easily interconvertible (Supplementary Fig. S11B).

We show that the external addition of several amino acids can partially rescue the phenotype of *cox11*, which is consistent with *cox11* having a globally altered metabolism in which some metabolites are lowly available, compromising the function of anaplerotic reactions required for the proper use of energy and resources.

## Discussion

### *Cox11* plants lack cytochrome c oxidase activity

This work reports the isolation and characterization of mutant lines of the moss *P. patens* knocked out for the conserved copper chaperone COX11, which is required for the insertion of copper ions into the active site of COX2 (Meyer et al. 2019). In other organisms, such as yeast, COX11 has been shown to be essential for the biogenesis of mitochondrial CIV (cytochrome c oxidase) with its depletion leading to a complete inactivation of the complex (Carr et al. 2002; Banting and Glerum 2006). Consistent with the sequence conservation across different organisms, *cox11* knockdown lines in Arabidopsis showed a strong reduction in CIV activity, but the complete KO was not viable (Radin et al. 2015).

In this work, we characterize plants completely depleted of COX11, whose encoding RNA was completely undetectable (Fig. 1; Supplementary Data Set S3). *cox11* plants have undetectable levels of cytochrome c oxidase activity, confirming that the protein is essential for CIV activity also in *P. patens*. Given the sensitivity of the spectroscopic quantification used (Fig. 1C), this ensures that residual cytochrome c oxidase activity, if any, is well below 5% of WT plants. To our knowledge, only 1 other plant mutant has been reported to have undetectable levels of cytochrome c oxidase, the *cod1* mutant in Arabidopsis (Dahan et al. 2014). However, *cod1* plants suffered from general and severe alterations and could only be analyzed by rescuing immature seeds, and analysis under physiological conditions was not possible (Dahan et al. 2014).

The isolation of a viable *cox11* mutant was instead possible in *P. patens* because this plant is photoautotrophic throughout its development, thus avoiding heterotrophic tissues, such as roots (Sakakibara et al. 2003). Mutants can be isolated by vegetative propagation under continuous light, bypassing sexual reproduction and spore formation/generation, thus avoiding life cycle stages where photosynthesis is not active and thus respiration is essential. In addition, *P. patens* cells are haploid during most of their life cycle, allowing the phenotype associated with any genetic modification to be directly assessed without reaching homozygosity. In *P. patens*, the effects of inactivation of mitochondrial respiration can thus be assessed in fully photosynthetically active cells, providing a highly valuable model for studying the physiological consequences of the constitutive blockade of the cyanide-sensitive respiratory pathway in plants.

In *cox11* plants, the cyanide-sensitive pathway is completely inactivated, and they largely rely on the AOX-mediated alternative pathway to oxidize ubiquinone and support mitochondrial electron transport, as demonstrated by the effect of the specific inhibitors (Fig. 3; Supplementary Fig. S5). The high electron flux capacity of the AOX pathway is not a feature induced by the mutation but rather a species-specific property, as *P. patens* WT plants also show strong respiratory activity even in the presence of cyanide (Fig. 3). This suggests that these plants have a high AOX electron transport capacity that is normally underutilized but can ensure sufficient electron transport activity in the absence of CIV. This hypothesis also explains why AOX has apparent increased activity without higher protein accumulation levels (Fig. 3). This observation is consistent with results from Arabidopsis plants with reduced levels of cytochrome c and CIV that also showed unchanged or even reduced levels of Aox1a, the homolog of Pp-Aox (Florez-Sarasa et al. 2021). Interestingly, this is different from results obtained with plants with altered CI activity, including *P. patens*, that commonly show AOX overaccumulation (Gutierres et al. 1997; Meyer et al. 2009; Fromm et al. 2016b; Pétriacq et al. 2017; Mellon et al. 2021). It should be mentioned that AOX overaccumulation in CI mutants has also been attributed to the induction of mitochondrial retrograde signaling rather than a rearrangement to maintain the ETR (Giraud et al. 2009; Millar et al. 2011). If this signaling pathway is not activated in *cox11*, this would also explain why this overaccumulation is not observed.

The *cox11* mutants show a rearrangement of respiratory apparatus with increased protein levels of NAD9, a core subunit of CI, and SDH1, the catalytic subunit of CII, suggesting an increased capacity for electrons to enter the modified respiratory chain, which, together with AOX, enabled the observed increased O_2_ consumption. This increased protein content was consistent with a general overexpression of several genes encoding for respiratory complexes, in particular several encoding for CI and CV (Supplementary Fig. S10). This expression was particularly induced during the day, after several hours of illumination, thus when photosynthesis was active. It is interesting to observe that genes encoding for CIV are also similarly regulated, suggesting these genes respond to the same signals stimulating the expression under illumination, even though there is no complex activity because of the lack of COX11.

### *Cox11* plants are not limited by energy availability

Photosynthetic electron transport produces NADPH in excess of ATP for CO_2_ fixation, and there are multiple mechanisms to balance this ratio, such as cyclic and pseudo-cyclic electron transport (Shikanai and Yamamoto 2017). Mitochondrial respiration is also a major contributor to metabolic regulation, consuming reducing power generated within the chloroplast that is transferred by metabolic shuttles (Burlacot and Peltier 2023) and converted to ATP (Vera-Vives et al. 2024). Several lines of evidence support the so-called chloroplast-to-mitochondrion electron flow contributes to optimal photosynthetic electron transport, as suggested by inhibitor experiments (Yoshida et al. 2006), modeling analyses (Shameer et al. 2019), and mutants in different species (Cardol et al. 2003; Dang et al. 2014; Bailleul et al. 2015; Mellon et al. 2021). As example, increasing the flow of reducing equivalents from chloroplasts to mitochondria by overexpressing the protein PAP2 had a positive effect in balancing the NADPH/ATP ratio in chloroplasts and increased photosynthetic efficiency in Arabidopsis (Voon et al. 2021).

*cox11* plants can be propagated vegetatively and are viable, but their growth is severely impaired. Remarkably, this is the case even if all protonema cells, used for all experiments, are fully photosynthetically active and even when plants are grown under 24 h of illumination, ensuring a constant supply of energy from photosynthesis and eliminating the potential effects of lack of energy during the night. This contrasts with respiratory mutants depleted in CIV in the green alga *C. reinhardtii*, which showed a growth delay only when grown under hetero/mixotrophic conditions, but were not significantly different from the WT in minimal medium when photosynthesis was active (Colin et al. 1995). This suggests that the role of respiration in photosynthetic organisms changed during plant evolution (Mellon et al. 2021).

It is interesting to observe that, differently from other plants with altered respiratory activity, *P. patens cox11* plants did not show a major alteration of photosynthetic electron transport reactions. At the same time, these previously analyzed mutants were all affected in CI, including those from *P. patens* that were analyzed in a similar manner as those presented here (Mellon et al. 2021). Since the organism and experimental setup are equivalent, this suggests that the different effect on photosynthetic ETR should be attributed to the different impact of CI and CIV mutations.

The NADH dehydrogenase complex (CI) is the main site for electron insertion into the mitochondrial electron transport chain (Braun et al. 2014). Its inactivation thus likely affects the mitochondria ability to consume reducing power from chloroplast. This decrease in reducing power consumption is expected to affect the photosynthetic electron transport and consistently, several evidence support the impact of CI inactivation on photosynthesis, impacting cell redox balance (Sabar et al. 2000; Noctor et al. 2007; Meyer et al. 2009; Hager et al. 2010) and with transcripts encoding the light reaction of photosynthesis downregulated in CI mutants (Pellny et al. 2008; Meyer et al. 2009). This hypothesis is also consistent with other data, such as proteome analyses, which showed that CI is more abundant in photosynthetically active tissues (Peters et al. 2012).

The absence of strong effects on the photosynthetic apparatus in the CIV mutant suggests that in these plants, the respiratory apparatus, also thanks to its rearrangements, is able to consume enough reducing power to balance photosynthetic activity, without inducing secondary regulation of the light phase of photosynthesis. The transfer of electrons from CI to molecular oxygen by AOX is less efficient than the cytochrome pathway in ATP production but can still ensure the consumption of reducing power exported from the chloroplast.

In any case, this hypothesis anyhow highlights that the consumption of reducing power from respiration is a major component of the functional interaction between chloroplasts and mitochondria and an essential function of respiration in photosynthetically active cells (Dahal et al. 2017; Krämer and Kunz 2021; Moreno-García et al. 2022). Following this line of reasoning, even if respiration is altered in CIV mutants, the effect on photosynthetic electron transport is less because they are able to maintain a sufficient rate of reducing power consumption.

Despite the mutation, *cox11* plants to maintain a similar or only slightly lower level of ATP through oxidative phosphorylation compared to the WT. This is supported by the observation that the relative AEC does not show a significant difference in *cox11* even at night (Fig. 7). Reduced *cox11* growth and consequent reduction in energy consumption could also contribute to keeping the AEC close to normal values. A similar effect has been shown in tobacco (*Nicotiana tabacum*) plants depleted in CI activity, where electron transport increased the efficiency of oxidative phosphorylation, so that predicted ATP production was maintained (Vidal et al. 2007). More recently, we similarly observed that in *P. patens* CI mutants increased chloroplast ATPase activity to maintain ATP levels (Mellon et al. 2021).

Despite the lower rates of carbon fixation, *cox11* plants also accumulate more starch, which is a product of photosynthesis, confirming that alterations in photosynthesis could not be the cause of the growth defects described in *cox11*. Eliminating photorespiration by growing plants under elevated CO_2_ also did not have any positive effect on growth. The cultivation under continuous illumination also did not rescue the phenotype, all observations that support the hypothesis that mutants are not experiencing an energy deficiency.

The synthesis of starch was also upregulated at the transcript level, by inducing several starch synthases during the day, again consistent with the idea that photosynthesis and carbon fixation activity were functional and probably in excess with respect to metabolic consumption. Since the amylolytic activity of the *cox11* extracts was higher than in WT, the observed overaccumulation cannot be explained by an impairment of starch mobilization, but rather by a limited ability to consume the products of its degradation. Since starch degradation is activated during the night, when mitochondrial electron transport is the main pathway for energy generation, it is likely that its impairment will affect sugar metabolization and consequently starch degradation, despite amylolytic activities being even induced. Consistent with this conclusion, higher starch accumulation has been reported for Arabidopsis mutants with decreased activity of the cyanide-sensitive pathway (Racca et al. 2018).

All these observations consistently point to the fact that photosynthesis and carbon fixation are active, at least enough to support the reduced growth of the plants. Cells are however largely limited by their ability to mobilize the reduced carbon available, as evidenced by the reduced levels of glycolytic intermediates at night when starch catabolism of starch should provide the cell with monosaccharides to fuel respiration.

Consistent with this hypothesis, attempts to rescue the growth phenotype by inducing a more favorable energetic state failed. The addition of glucose and ammonium tartrate had a positive effect on growth, but this was the same as in WT and the large growth defect of the mutant was maintained. Neither additional light of any duration or intensity nor increased CO_2_ supply was able to rescue the phenotype, suggesting that alterations in photosynthesis, CO_2_ fixation, or photorespiration, even if affected by the mutation, were not the main responsible for growth impairment. In summary, *cox11* growth defects could not be attributed to a simple energy deficit, but rather to an insufficient capacity to utilize the reduced carbon synthesized thanks to photosynthesis.

### Mitochondrial metabolism has an essential impact on plant amino acid biosynthesis

Based on the conclusions above, the analysis of CIV mutants is thus suitable to evidence other biological functions of mitochondrial respiration in plants beyond the interaction with photosynthesis. Blocking respiration had a major impact on amino acid metabolism, as evidenced by both transcriptomic and metabolomic data. Glycolysis and the TCA cycle are pivotal for the plant cell as they provide not only readily available energy in the form of nucleotide phosphates such as ATP and GTP but also metabolic intermediates for anaplerotic reactions (Sweetlove et al. 2010; Zhang et al. 2018). For example, glutamate, found at lower levels at night in *cox11*, is an important bridge between carbon and nitrogen metabolism because it can be reversibly converted to the TCA intermediate 2-ketoglutarate (Hodges 2002; Forde and Lea 2007). The shortage of glycolysis and TCA cycle intermediates is therefore expected to alter the processes of nitrogen assimilation and mobilization. Indeed, nitrate assimilation in the leaves of vascular plants can occur at night by using stored carbohydrates (Yoneyama and Suzuki 2020).

Ammonium assimilation is done mainly through the glutamine synthetase/GOGAT (GS/GOGAT) cycle, although it could also possibly be assimilated via glutamate dehydrogenase. The GS/GOGAT cycle requires glutamate, glutamine, and 2-ketoglutarate, all of which were found at lower levels in *cox11* at night (Gaufichon et al. 2010; Yoneyama and Suzuki 2020). Nitrogen assimilation proceeds in part through the amination of aspartate and glutamate into asparagine and glutamine, respectively. The absolute values of the aminated forms of asparagine and glutamine were lower, but also the aspartate/asparagine and glutamate/glucose ratios were much higher in *cox11*, especially during the day; that is, the deaminated forms of the amino acids were predominant, suggesting that nitrogen assimilation was not efficient (Supplementary Fig. S11).

These alterations in amino acid content and biosynthetic pathways are consistent with the growth phenotype of *cox11* plants in supplemented media (Fig. 8). Even if there was no optimization of the concentrations employed, amino acid treatments generally had beneficial or at least neutral effects on *cox11*, even in cases where they had a toxic effect on WT growth, a phenomenon commonly reported in plants (Bonner et al. 1992; Forsum et al. 2008). In some cases, the external supply of amino acids showed a particularly strong improvement in growth and significant rescue of the gap with WT. This is true for asparagine, glutamine, and serine, 3 amino acids detected in lower levels at ZT0 and ZT2 in *cox11*. The most significant improvement was achieved when plants were grown in the presence of the amino acid serine (Fig. 8), whose positive effect is not attributable to its involvement in photorespiration, since growth under high CO_2_ did not have a positive effect on *cox11* plants.

Therefore, these data suggest that respiration plays a key role at the interface between carbon and nitrogen metabolism. When inactivated, cells cannot support the effective incorporation of carbon skeletons into amino acids, severely impairing their biosynthesis and thus having a major negative impact on growth.

## Materials and methods

### Plant material and growth conditions

*P. patens* (Gransden ecotype) was amplified through vegetative propagation as done previously on solid PpNH_4_ medium (Mellon et al. 2021). If not stated diversely, physiological and biochemical characterizations were performed on 10-d-old tissue cultivated in PpNO_3_ medium (Mellon et al. 2021) at 22 °C and a long-day photoperiod (light:dark 16 h:8 h).

### Generation of *COX11* KO lines

For generation of *COX11* KO lines, up- and downstream regions of the locus harboring the *COX11* gene were cloned into a BHRf plasmid (Alboresi et al. 2010), which carries a hygromycin resistance cassette (Supplementary Fig. S1, A and C). The construct was linearized with the restriction enzyme PvuII (Thermo Fisher Scientific) and used for gene targeting through PEG-mediated transformation as described previously (Mellon et al. 2021). Genomic DNA (gDNA) was extracted from stably resistant clones following a fast extraction protocol with some modifications (Edwards et al. 1991), and PCR amplifications of recombination cassette were performed on extracted gDNA. Protonemata of *P. patens* grown on PpNH_4_ were homogenized in 2-mL tubes using 3-mm zirconium glass beads (Merck) in the presence of 500 *µ*L of cold TEN buffer (Tris-HCl 100 mm, pH 8.0; EDTA 50 mm; NaCl 500 mm). After the addition of 35 *μ*L of SDS 20%, samples were incubated at 65 °C for 5 min. Then, 130 *μ*L of potassium acetate 5 m were added, and samples were kept on ice for 5 min and centrifuged at 4 °C for 10 min at 13,000 × *g*. The supernatant was transferred to a clean 1.5-mL tube containing 500 *μ*L of isopropanol at −20 °C, mixed by inversion, and incubated at −20 °C for 10 min. Then, a series of centrifuges at 4 °C and 13,000 × *g* were performed. Supernatant was discarded after a first centrifuge of 10 min, and the pellet was suspended in 500 *μ*L of ethanol 70%. The resultant pellet of a second centrifuge of 5 min was suspended in 150 *μ*L of ethanol 70%. A last centrifuge of 2 min was performed, and the resultant pellet was dried off under the chemical fume hood and suspended in 50 *μ*L of water. This DNA solution was kept at −20 °C until used.

To confirm that *cox11* lines lacked the *COX11* expression, RT-PCR was performed on cDNA using RevertAid reverse transcriptase (Thermo Fisher Scientific) synthesized after RNA extraction. Primers used for construct design and line validation are included in Supplementary Table S3. CIV activity was quantified spectrophotometrically, as previously described (Birch-Machin et al. 1994). In brief, 2 *µ*L of crude membrane extracts (Pineau et al. 2008) were added to the reaction mixture containing 0.09 m phosphate buffer and 8 mm reduced cytochrome c. Control was prepared adding 5-*µ*L KCN 0.05 m to WT extracts. The CIV-specific activity was quantified by the decrease of OD_550 nm_, the specific absorbance of reduced cyt c, an extinction coefficient ε_550_ = 27.7 mm^−1^ cm^−1^ and normalized to the total protein content. Three independent lines, named #148, #150, and #152, were used in the experiments reported in this article. Each experiment contains data obtained from at least 2 independent lines.

### Public transcriptomic data and sequence alignment

The expression levels of *COX11* in different tissues and conditions were compared using the publicly available data from PeatMOSS (Fernandez-Pozo et al. 2020). For comparison of conservation degree of COX11, the protein sequences from cattle (*B. taurus*), UniProt ID A3KMZ6; yeast (*S. cerevisiae*), UniProt ID P19516; and *P. patens*, UniProt ID A0A2K1J6Q0_PHYPA were retrieved and then aligned using Clustal Omega v. 1.2.4 (Sievers et al. 2011).

### Western blot analysis

Protein extraction and immunoblotting were performed as described previously (Mellon et al. 2021). Samples were loaded so that the same chlorophyll amount was present. For chlorophyll quantification, 2 mL of pure protein extracts were diluted in 68 *μ*L of acetone 80% in 0.5 mL tubes. The tubes were vortexed briefly and centrifuged at 13,000 × *g* for 5 min at room temperature. Proteins precipitated forming a pellet and chlorophylls remained in solution. The supernatant was transferred to clean 0.5-mL tubes. The absorption spectrum (from 750 to 600 nm) of the supernatant was measured using a Cary 100 UV-Vis spectrophotometer (Agilent Technologies). The chlorophylls concentration of the pure protein extract, [*Chl*], was calculated applying the following formula, where OD stands for optical density (Porra et al. 1989):


$$\begin{array}{rl} {}[Chl]\left(\frac{\mu g}{\mathrm{mL}}\right)\;= & \left\{20.20\cdot [\mathrm{OD}(645)-\mathrm{OD}(750)]+8.02\cdot [\mathrm{OD}(663\right)\\ & -\mathrm{OD}(750)]\}. \end{array}$$

After quantification, the pure protein extracts were incubated 1 min at 100 °C.

The list of antibodies used is attached in Supplementary Table S4. For all the antibodies except for the ß subunit of CV, total protein extracts were resolved by SDS-PAGE. For the ß subunit of CV, crude mitochondria extracts were separated by Urea-PAGE.

### Crude membrane isolation, blue native protein electrophoresis (BN-PAGE) and CIV activity staining

Crude membrane extracts were prepared on ice and using chilled tubes and reagents to avoid protein denaturation following a protocol adapted after (Pineau et al. 2008). Approximately 300 mg of fresh or frozen (−80 °C) protonema grown on PpNO_3_ for 10 d were homogenized using a Potter–Elvehjem glass tissue grinder in 2 mL of MOPS-KOH 75 mm, pH 7.6; sucrose 0.6 m; EDTA 4 mm; PVP-40 0.2%; cysteine 8 mm; and bovine serum albumin 0.2%. The homogenate was transferred to a 15-mL conical tube lined with a square of Miracloth with 20-*μ*m pores to filter unbroken cells and tissue debris. The filtered homogenate was then transferred to a 2-mL tube and centrifuged for 4 min at 1,300 × *g* to pull down the cellular debris. The supernatant was collected in a clean 2-mL tube and centrifuged for 20 min at 21,470 × *g* to pellet the thylakoid and mitochondrial membranes. The supernatant was discarded, and the pellet was resuspended in 200 *μ*L of MOPS-KOH pH 7.2 and sucrose 0.3 m. Total protein content was determined by the bicinchoninic acid (BCA) assay.

A 4% to 12% running acrylamide gradient polyacrylamide gel was freshly prepared, with a stacking 4% gel. Protein solubilization protocol was adapted after (Pineau et al. 2008). Twenty microliters of digitonin 4% (*w*/*v*) prepared in ACA buffer (1.5 m aminocaproic acid, 0.1 m Bis-Tris-HCl pH 7.0, and 2 mm EDTA) were added to the tubes containing 20 *µ*L of crude extracts in ACA buffer that corresponded to 50 *µ*g of proteins, reaching 2% digitonin in ACA buffer. Each tube was incubated on ice for 20 min and centrifuged at 4 °C at 22,000 × *g* for 8 min. The supernatant containing solubilized protein complexes was transferred to a clean tube, supplemented with 4 *µ*L of Coomassie blue 5% solution (20 mm Bis-Tris, 0.5 m aminocaproic acid, and Coomassie blue G-250 5% [*w*/*v*]) and loaded to the gel.

Sample loading and gel running were performed in a cold chamber at 4 °C according to an adapted version of the protocol described by Järvi et al. (2011). The electrode assembly with the mounted gel was filled with both cathode buffer (15 mm Bis-Tris-HCl, pH 7.0; 50 mm tricine) containing Coomassie blue G-250 0.02% (*w*/*v*) and anode buffer (50 mm Bis-Tris-HCl, pH 7.0). The wells were loaded with the solubilized samples using a Hamilton syringe. The gel was run at 75 V for 30 min. Then, the cathode buffer was replaced with fresh cathode buffer without Coomassie blue, and the gel was run at 100 V for 30 min, at 125 V for 30 min, at 150 V for 60 min, at 175 V for 30 min, and at 200 V for 60 min. The total running time was about 4 h. After running, the gel was kept at 4 °C until used for in-gel activity staining.

After running, in-gel activity staining was done as described in Sabar et al. (2000). The gel was incubated for 1 h in CIV buffer (50 mm potassium phosphate buffer pH 7.4; 75 mg/mL sucrose). Then, the buffer was replaced with CIV staining solution (1 mg/mL 3,3′-diaminobenzidine tetrahydrochloride hydrate [Merck D5637]; 1 mg/mL cytochrome c [Merck C7752] in CIV buffer). The gel was incubated in the staining solution for 24 h at room temperature before image acquisition.

### In-gel amylolytic activity staining

For in-gel amylolytic activity, proteins were extracted 1:2 (fresh weight:volume) in 100 mm MOPS pH 7.2, 1 mm EDTA, 10% glycerol, 5 mm DTT, and 1 mm phenylmethylsulfonyl fluoride using a pestle. Proteins were quantified by the BCA assay. For each sample, 10 *µ*g were mixed to native loading buffer (60 mm Tris-HCl pH 6.8; 10% glycerol; 0.025% bromophenol blue) and loaded into a 7.5% acrylamide gel containing 0.1% solubilized amylopectin from potato starch (Merck). Gels were run at 120 V for 4 h at 4 °C and then incubated in incubation buffer (100 mm Tris-HCl pH 7; 1 mm MgCl_2_; 1 mm CaCl_2_; 2 mm DTT) for 15 min, and the buffer was changed with new buffer and incubation left overnight at room temperature or at 37 °C for 4 h. After the incubation, gels were washed with bidistilled water and stained with Lugol's solution (0.33% I_2_, 0.66% KI).

### Growth test

Two-millimeter disks of protonema of *P. patens* grown for 10 d in long-day conditions were distributed in 92-mm Petri dishes filled with either solid PpNO_3_, PpNH_4_, or amino acid-enriched media. A cellophane filter was not added as routinary done for other experiments. Each Petri dish contained 10 plant disks, and at least 2 technical replicates per genotype were included. The space distribution of the disks on the plate was homogenous, and the relative position of each disk was aleatory, but the same loading scheme was followed for the different conditions to be compared. Colony size was measured periodically. Photographs of the plates were acquired using a smartphone. Images were processed with Fiji (Schindelin et al. 2012) to remove the plate background using the “Threshold Colour” plugin and/or the “Threshold…” utility after image conversion to 8 bits. Scale was set using the diameter of the Petri dish (92 mm), and area in mm^2^ was measured for each colony.

The effect of photoperiod was assessed by growing identical plates under either long-day or continuous illumination (24-h light). The effect of high CO_2_ was evaluated by keeping the Petri dishes in a growth chamber with controlled atmosphere of 1% CO_2_ in continuous illumination and 22 °C. The effect of amino acid addition was tested by performing the growth test on solid PpNO_3_ medium containing an additional 3 mm of each of the 20 primary amino acids except for tyrosine, which could not be dissolved without strongly acidifying the medium. The final concentration of 3 mm was chosen according to previous experiments done in Arabidopsis (Forsum et al. 2008).

### Measurement of oxygen consumption and evolution

Measurements of oxygen consumption (respirometry) and oxygen evolution were performed on pieces of intact protonema from 10-d-old plants grown on PpNO_3_ and dark adapted for 40 min before the experiments. Measurements were performed using a test version of the NextGen-O2k and the PhotoBiology (PB)-Module (Oroboros Instruments, Innsbruck) with the software DatLab 7.4.0.4 (Went et al. 2021). The PB light source contained a blue OSLON LED (emitting wavelength range 439 to 457 nm with the peak at 451 nm) attached to the window of the NextGen-O2k chamber. The oxygen concentration was assessed in 2-mL measuring chambers at 22 °C with a 2-s frequency, and samples were magnetically stirred at 750 rpm.

To avoid disruption of the moss samples during the measurement, we used a sample holder developed by Oroboros Instruments, Innsbruck. At first, we filled the measuring chambers with a volume slightly higher than 2 mL of fresh and sterile PpNO_3_ medium containing 10 mm NaHCO_3_ (to avoid carbon limitation during photosynthetic measurements), and we inserted the sample holder and closed the chamber removing excess volume to ensure that precise 2 mL were inside. Then, we moved the stopper to the open position and let the system equilibrate for a few minutes with the stirring on. This served both to bring the medium to the experimental temperature and to equilibrate the oxygen concentration of the medium to the atmospheric oxygen values. We opened the chamber, added a piece of protonema of approximately 1 cm^2^ on top of the sample holder, and closed the chamber. This operation was done minimizing the exposure of moss samples to light. Oxygen concentration in the chamber was monitored for 10 min at dark to assess the rate of dark respiration, and we proceeded either with the quantification of photosynthesis or with the assessment of the effect of respiratory inhibitors.

For quantifying oxygen evolution, after stabilization of the respiration signal, blue light was turned on at 500 *μ*mol photons m^−2^ s^−1^, which was well above the saturating levels for *P. patens*. We kept the sample under this illumination for 10 min to achieve the stabilization of the oxygen evolution rate. The values of oxygen evolution rate reported in this work correspond to the median of 40 to 50 points in the stable region of oxygen flux. The plot of a representative experiment is shown in Supplementary Fig. S8.

For quantifying the effect of inhibitors on dark respiration, after stabilization of the respiration signal, inhibitors were added sequentially to the chamber. The plot of a representative experiment is shown in Supplementary Fig. S6. Inhibitors were added through the stopper using Hamilton syringes, therefore not interrupting the measurements. For titrations using KCN or antimycin A, we added to the chamber 4 *µ*L of a stock 500 mm; for titrations using SHAM or n-propylgallate, we added to the chamber 8 *µ*L of a stock 250 mm. In all cases, the final concentration of the inhibitor was 1 mm.

After each experiment, the moss sample was recovered from the chamber and used for chlorophyll quantification as described earlier in this section. The measure of oxygen consumption and evolution was therefore normalized to the amount of chlorophylls in the sample.

### Gas exchange measurement

The CO_2_ assimilation rate was measured with the LI-6800 portable photosynthetic system (LI-COR Biosciences, USA) in 8-wk-old gametophytes of WT and 16-wk-old gametophytes of *cox11* mutants. Gametophytes were cultivated on the PPNO_3_ medium with a glass filter to separate them easier from the medium after measurement to obtain accurate dry weight. Gametophytes with medium block were placed in the bryophyte chamber (part no.: 6800-24) with the large light source (part no.: 6800-03). All measurements were made at an air humidity of 80% to 85%, CO_2_ concentration of 400 ppm, and chamber temperature of 25 °C. After gas exchange measurements, the CO_2_ assimilation rate was recalculated by dry weight. The light response curve was performed after 10-min light induction at 300 *μ*mol photons m^−2^ s^−1^. For each light intensity (600, 400, 250, 150, 100, 50, 20, and 0 *μ*mol photons m^−2^ s^−1^), the CO_2_ assimilation rate was logged upon reaching a steady condition after 2 min.

### Spectroscopic analyses

In vivo Chl fluorescence analyses were performed as described previously (Mellon et al. 2021).

### Transmission electron microscopy

Ten-day-old plants grown under long day were kept at dark for circa 20 h before fixation, to avoid overaccumulation of starch. Small pieces of samples (about 2 to 3 mm^3^) were fixed with 3.0% glutaraldehyde (EMS 16220) plus 1% paraformaldehyde (Merck P6148) plus 0.5% tannic acid in 0.05 m sodium cacodylate buffer pH 7.4 for 2 h at room temperature. Subsequently, the samples were postfixed with 1% in 0.1 m sodium cacodylate buffer for 1 h at 4 °C. After 3 water washes, samples were dehydrated in a graded ethanol series and embedded in an epoxy resin (Sigma-Aldrich 46345). Ultrathin sections (60 to 70 nm) were obtained with a Leica Ultracut EM UC7 ultramicrotome, counterstained with uranyl acetate and lead citrate, and viewed with a Tecnai G2 (FEI) transmission electron microscope operating at 100 kV. Images were captured with a Veleta (Olympus Soft Imaging System) digital camera.

### Sample harvesting for systems analysis

We optimized the following system for growing moss samples for systems level analyses, i.e. RNA-seq and untargeted metabolomics (Supplementary Fig. S12). Protonema grown for 1 wk on solid PpNH_4_ medium under continuous illumination was disrupted using a T 25 UltraTurrax (IKA) and used for the inoculation of a 100-mL Erlenmeyer flask with 20 mL of liquid PpNH_4_. After 1 wk of growth under continuous illumination, the plant was harvested, disrupted, and used for the inoculation of a 250-mL Erlenmeyer flask with 50 mL of liquid PpNO_3_. After 1 wk of growth under continuous illumination, the plant was harvested, disrupted, and used for the inoculation of a 500-mL Erlenmeyer flask with 100 mL of liquid PpNO_3_. After 1 wk of growth, the plant material was distributed into a layer of Miracloth arranged on top of a plastic cylinder, inside of a Magenta box filled with fresh liquid PpNO_3_. The plant was in contact with the medium through the filter, allowing growth under this “hydroponics” system. Plants in closed Magenta boxes were grown for 15 d under a short-day regime (light:dark 12 h:12 h). The day of harvesting, plants were immediately snap frozen at the corresponding ZT (ZT0, ZT2, and ZT6). For plants harvested at ZT0, Magenta boxes were enclosed in aluminum foil at the beginning of the night period (ZT12), and samples were snap frozen at ZT0 in a dark room illuminated with dim, green light. For plants harvested at ZT2 or ZT6, plants were snap frozen directly in the growth chamber, avoiding shading the plant until it had been frozen. Frozen samples were kept at −80 °C until used.

### RNA-seq, differential gene expression analysis, and pathway enrichment analysis

Frozen samples were powdered using a cold mortar and pestle, in the presence of liquid nitrogen. Approximately 150 mg of powder were used for RNA extraction using the RNeasy Plant Mini Kit, ref. 74904 (Qiagen). RNA was used to build cDNA libraries using the kit QuantSeq 3′ mRNA-Seq Library Prep Kit FWD (Lexogen), which exploits oligo-d(T) primers therefore enriching the cDNA library in cDNA representative of mRNA, but not ribosomal RNA or other types of RNA. Libraries were then sequenced with a depth of 5 million of reads using a NextSeq 500 System (Illumina).

The reference genome (Physcomitrium patens v3.3) from the JGI Plant Gene Atlas (Lang et al. 2018) was indexed using Bowtie 2 (v2.2.5) (Langmead and Salzberg 2012). Reads were then aligned using Bowtie 2, and bam files were generated with SAMtools (v 1.6) (Danecek et al. 2021). HTSeq-count (v 2.0.2) (Putri et al. 2022) was used to align the mapped reads to the genes provided in the annotation file (Ppatens_318_v3.3.gene.gff).

Differential expression analysis was done through the tool iDEP (v 0.96) (Ge et al. 2018). First, a low filter was applied to the gene list to keep only genes with at least 50 reads in at least 1 sample, which were 16,101. Then, their expression values of were log_2_ transformed, applying the formula *x*′ = log_2_(*x* + 1). Differential expression was analyzed using the integrated *limma* package (Ritchie et al. 2015), setting the false discovery rate cutoff at 0.05 and the fold-change cutoff at 2. The lists of DEGs at each condition are given in Supplementary Data Set S1. These lists were used for pathway enrichment analysis with iDEP using all the annotation databases available for *P. patens* in iDEP, which were based in gene orthology, or annotated protein domains coming from different sources (UniProt, InterPro, Pfam, and SMART). The lists and composition of enriched pathways at different conditions are given in Supplementary Data Set S2.

The list of genes encoding for ascorbate peroxidase was retrieved from 3 different sources. Some of them had alternative names in different publications. The summarized information is included in Supplementary Table S5. The list of genes encoding for superoxide dismutases and glutathione reductases was retrieved from Higashi et al. (2013) and Xu et al. (2013). For glutathione reductase, the 5 genes defined as H_2_O_2_ responsive by Liu et al. (2013) were included.

### Untargeted metabolomics and integrated pathway analysis

To a 1.5-mL tube containing 50 to 100 mg of powdered, frozen moss samples, we added 350 *µ*L of extraction solution (chloroform:methanol 10:4.28) and vortexed and incubated at −20 °C for 1 h. Then, we added 560 *µ*L of internal standard stock solution, kept on ice for 5 min with frequent vortexing, and centrifuged for 2 min at 20,000 × *g*. We then collected the supernatant into fresh 2-mL tubes and performed a second extraction of the remaining organic phase by adding 260 *µ*L of H_2_O, followed by incubating on ice for 5 min with frequent vortexing and centrifugation for 2 min at 20,000 × *g*. The second supernatant was added to the previous one. The extracts were kept at −80 °C until analysis by 2 different methods: GC-MS (Shim et al. 2020) and IC-MS (Curien et al. 2021). Since we produced 2 or 3 biological replicas for each genotype for each condition, we combined the replicas from the 2 independent *cox11* lines into a broader group that contained 4 to 6 replicas per each condition. This increases the power of statistical analyses. The abundance of metabolites was normalized to the internal standard and to the dry weight.

We then used the data as input values for the publicly available tool MetaboAnalyst (Xia and Wishart 2011) and used the graphics user interface to generate volcano plots and compare metabolite levels between mutant and WT at the different ZTs. As an output, we obtained the log-fold change values and *P*-values of every single comparison. To elaborate lists of metabolites that were significantly accumulated or depleted at a given condition, we established the significance threshold at *P* < 0.1. Heatmap in Fig. 6 was obtained using the platform SRPlot (Tang et al. 2023).

AEC was calculated as (ATP + ½ ADP)/(ATP + ADP + AMP) according to Tyutereva et al. (2022), based on the relative concentration of ATP, ADP, and AMP obtained by IC-MS after normalization to the internal standard thio-ATP.

Integrated pathway analysis was done by visualizing transcriptomic and metabolomic data with Pathway Tools (Karp et al. 2021) using the MossCyc v8.0.2 database from the Plant Metabolic Network (Hawkins et al. 2021). Metabolic diagrams in Fig. 6B were manually produced.

### Quantification of starch

Quantification of starch was done on total extracts of 10-d-old *P. patens* samples following the protocol described in Smith and Zeeman (2006).

### Accession numbers

Accession numbers of gene sequences are as follows: Cox11, Pp3c16_1230; Actin7, Pp3c3_33440. Accession numbers of specific pathways are included in Supplementary Tables S1, S2, and S5 and Fig. S10. Raw sequences have been submitted to the Sequence Read Archive (NCBI) under the project PRJNA1219350.

## Supplementary Material

koaf101_Supplementary_Data

## Data Availability

The data underlying this article will be shared on reasonable request to the corresponding author.

## Supplementary data

The following materials are available in the online version of this article.

**Supplementary Figure S1.** Multiple sequence alignment of Cox11 from *P. patens* and its homologs in yeast (*S. cerevisiae*) and cattle (*B. taurus*).

**Supplementary Figure S2.** Expression levels of *COX11* at different developmental stages.

**Supplementary Figure S3.** The *COX11* locus was used to produce the KO mutant lines and quantify CIV activity.

**Supplementary Figure S4.** Differential development of gametophores in *cox11*.

**Supplementary Figure S5.** Effect of antimycin and n-propylgallate on dark the respiration of intact protonema.

**Supplementary Figure S6.** Immunoblotting against the photosynthetic machinery subunits.

**Supplementary Figure S7.** Net photosynthesis in intact protonema.

**Supplementary Figure S8.** RNA-seq data overview and enriched pathways segregated for the 3 ZTs tested.

**Supplementary Figure S9.** Bar plots showing the most significant pathways that are differentially expressed in *cox11*.

**Supplementary Figure S10.** Ratios comparing the relative abundances of glutamate/glutamine and aspartate/asparagine.

**Supplementary Figure S11.** Effect of different amino acids on *cox11* growth.

**Supplementary Figure S12.** Protocol followed to amplify moss material for transcriptomics and metabolomics.

**Supplementary Table S1.** Relative expression of genes induced during UPR^ER^ in *P. patens*.

**Supplementary Table S2.** Relative expression of antioxidant enzymes in *cox11*.

**Supplementary Table S3.** Primers used in this work.

**Supplementary Table S4.** Antibodies used in this work.

**Supplementary Table S5.** Available information on ascorbate peroxidase-encoding genes in *P. patens*.

**Supplementary Data Set S1.** List of DEGs in *cox11* at different ZTs.

**Supplementary Data Set S2.** List and gene composition of the enriched pathways in *cox11* at different ZTs.

**Supplementary Data Set S3.** Relative expression of genes encoding respiratory complexes in *P. patens*.

**Supplementary Data Set S4.** List of identified metabolites and their relative levels in *cox11* at different ZTs.

## Dive Curated Terms

The following phenotypic, genotypic, and functional terms are of significance to the work described in this paper:

PAP2 Gramene: AT4G29080
PAP2 Araport: AT4G29080

ATP CHEBI: CHEBI:15422

PGM Gramene: AT5G51820
PGM Araport: AT5G51820

TPI Gramene: AT3G55440
TPI Araport: AT3G55440

## References

[koaf101-B1] Alboresi A, Gerotto C, Giacometti GM, Bassi R, Morosinotto T. *Physcomitrella patens* mutants affected on heat dissipation clarify the evolution of photoprotection mechanisms upon land colonization. Proc Natl Acad Sci U S A. 2010:107(24):11128–11133. 10.1073/pnas.1002873107 20505121 PMC2890724

[koaf101-B2] Alric J, Johnson X. Alternative electron transport pathways in photosynthesis: a confluence of regulation. Curr Opin Plant Biol. 2017:37(i):78–86. 10.1016/j.pbi.2017.03.014 28426976

[koaf101-B3] Aoyama T, Hiwatashi Y, Shigyo M, Kofuji R, Kubo M, Ito M, Hasebe M. AP2-type transcription factors determine stem cell identity in the moss *Physcomitrella patens*. Development. 2012:139(17):3120–3129. 10.1242/dev.076091 22833122

[koaf101-B4] Atkinson DE . The energy charge of the adenylate pool as a regulatory parameter. Interaction with feedback modifiers. Biochemistry. 1968:7(11):4030–4034. 10.1021/bi00851a033 4972613

[koaf101-B5] Bailleul B, Berne N, Murik O, Petroutsos D, Prihoda J, Tanaka A, Villanova V, Bligny R, Flori S, Falconet D, et al Energetic coupling between plastids and mitochondria drives CO_2_ assimilation in diatoms. Nature. 2015:524(7565):366–369. 10.1038/nature14599 26168400

[koaf101-B6] Banting GS, Glerum DM. Mutational analysis of the *Saccharomyces cerevisiae* cytochrome c oxidase assembly protein Cox11p. Eukaryot Cell. 2006:5(3):568–578. 10.1128/ec.5.3.568-578.2006 16524911 PMC1398067

[koaf101-B7] Beheshti H, Strotbek C, Arif MA, Klingl A, Top O, Frank W. PpGRAS12 acts as a positive regulator of meristem formation in *Physcomitrium patens*. Plant Mol Biol. 2021:107(4–5):293–305. 10.1007/s11103-021-01125-z 33598827 PMC8648639

[koaf101-B8] Birch-Machin MA, Briggs HL, Saborido AA, Bindoff LA, Turnbull DM. An evaluation of the measurement of the activities of Complexes I–IV in the respiratory chain of human skeletal muscle mitochondria. Biochem Med Metab Biol. 1994:51(1):35–42. 10.1006/bmmb.1994.1004 8192914

[koaf101-B9] Bonner CA, Rodrigues AM, Miller JA, Jensen RA. Amino acids are general growth inhibitors of *Nicotiana silvestris* in tissue culture. Physiol Plant. 1992:84(3):319–328. 10.1111/j.1399-3054.1992.tb04671.x

[koaf101-B10] Boursiac Y, Boudet J, Postaire O, Luu D-T, Tournaire-Roux C, Maurel C. Stimulus-induced downregulation of root water transport involves reactive oxygen species-activated cell signalling and plasma membrane intrinsic protein internalization. Plant J. 2008:56(2):207–218. 10.1111/j.1365-313X.2008.03594.x 18573191

[koaf101-B11] Braun H-P, Binder S, Brennicke A, Eubel H, Fernie AR, Finkemeier I, Klodmann J, König A-C, Kühn K, Meyer E, et al The life of plant mitochondrial Complex I. Mitochondrion. 2014:19(Pt B):295–313. 10.1016/j.mito.2014.02.006 24561573

[koaf101-B12] Burlacot A, Peltier G. Energy crosstalk between photosynthesis and the algal CO_2_-concentrating mechanisms. Trends Plant Sci. 2023:28(7):795–807. 10.1016/j.tplants.2023.03.018 37087359

[koaf101-B13] Cardol P, Gloire G, Havaux M, Remacle C, Matagne R, Franck F. Photosynthesis and state transitions in mitochondrial mutants of *Chlamydomonas reinhardtii* affected in respiration. Plant Physiol. 2003:133(4):2010–2020. 10.1104/pp.103.028076 14630958 PMC300752

[koaf101-B14] Cardol P, González-Halphen D, Reyes-Prieto A, Baurain D, Matagne RF, Remacle C. The mitochondrial oxidative phosphorylation proteome of *Chlamydomonas reinhardtii* deduced from the genome sequencing project. Plant Physiol. 2005:137(2):447–459. 10.1104/pp.104.054148 15710684 PMC1065347

[koaf101-B15] Carr HS, George GN, Winge DR. Yeast Cox11, a protein essential for cytochrome *c* oxidase assembly, is a Cu(I)-binding protein. J Biol Chem. 2002:277(34):31237–31242. 10.1074/jbc.M204854200 12063264

[koaf101-B16] Cavalcanti JHF, Quinhones CGS, Schertl P, Brito DS, Eubel H, Hildebrandt T, Nunes-Nesi A, Braun H-P, Araújo WL. Differential impact of amino acids on OXPHOS system activity following carbohydrate starvation in Arabidopsis cell suspensions. Physiol Plant. 2017:161(4):451–467. 10.1111/ppl.12612 28767134

[koaf101-B17] Chamizo-Ampudia A, Sanz-Luque E, Llamas A, Galvan A, Fernandez E. Nitrate reductase regulates plant nitric oxide homeostasis. Trends Plant Sci. 2017:22(2):163–174. 10.1016/j.tplants.2016.12.001 28065651

[koaf101-B18] Chellamuthu V-R, Ermilova E, Lapina T, Lüddecke J, Minaeva E, Herrmann C, Hartmann MD, Forchhammer K. A widespread glutamine-sensing mechanism in the plant kingdom. Cell. 2014:159(5):1188–1199. 10.1016/j.cell.2014.10.015 25416954

[koaf101-B19] Colin M, Dorthu M-P, Duby F, Remacle C, Dinant M, Wolwertz M-R, Duyckaerts C, Sluse F, Matagne RF. Mutations affecting the mitochondrial genes encoding the cytochrome oxidase subunit I and apocytochrome b of *Chlamydomonas reinhardtii*. Mol Gen Genet. 1995:249(2):179–184. 10.1007/BF00290364 7500939

[koaf101-B20] Curien G, Lyska D, Guglielmino E, Westhoff P, Janetzko J, Tardif M, Hallopeau C, Brugière S, Dal Bo D, Decelle J, et al Mixotrophic growth of the extremophile *Galdieria sulphuraria* reveals the flexibility of its carbon assimilation metabolism. New Phytol. 2021:231(1):326–338. 10.1111/nph.17359 33764540 PMC8252106

[koaf101-B21] Dahal K, Martyn GD, Alber NA, Vanlerberghe GC. Coordinated regulation of photosynthetic and respiratory components is necessary to maintain chloroplast energy balance in varied growth conditions. J Exp Bot. 2017:68(3):657–671. 10.1093/jxb/erw469 28011719 PMC5441918

[koaf101-B22] Dahan J, Tcherkez G, Macherel D, Benamar A, Belcram K, Quadrado M, Arnal N, Mireau H. Disruption of the CYTOCHROME C OXIDASE DEFICIENT1 gene leads to cytochrome c oxidase depletion and reorchestrated respiratory metabolism in Arabidopsis. Plant Physiol. 2014:166(4):1788–1802. 10.1104/pp.114.248526 25301889 PMC4256860

[koaf101-B23] Danecek P, Bonfield JK, Liddle J, Marshall J, Ohan V, Pollard MO, Whitwham A, Keane T, McCarthy SA, Davies RM, et al Twelve years of SAMtools and BCFtools. GigaScience. 2021:10(2):giab008. 10.1093/gigascience/giab008 33590861 PMC7931819

[koaf101-B24] Dang K-V, Plet J, Tolleter D, Jokel M, Cuiné S, Carrier P, Auroy P, Richaud P, Johnson X, Alric J, et al Combined increases in mitochondrial cooperation and oxygen photoreduction compensate for deficiency in cyclic electron flow in *Chlamydomonas reinhardtii*. Plant Cell. 2014:26(7):3036–3050. 10.1105/tpc.114.126375 24989042 PMC4145130

[koaf101-B25] De Col V, Fuchs P, Nietzel T, Elsässer M, Voon CP, Candeo A, Seeliger I, Fricker MD, Grefen C, Møller IM, et al ATP sensing in living plant cells reveals tissue gradients and stress dynamics of energy physiology. eLife. 2017:6:e26770. 10.7554/eLife.26770 28716182 PMC5515573

[koaf101-B26] Edwards K, Johnstone C, Thompson C. A simple and rapid method for the preparation of plant genomic DNA for PCR analysis. Nucleic Acids Res. 1991:19(6):1349. 10.1093/nar/19.6.1349 2030957 PMC333874

[koaf101-B27] Esposti MD . On the evolution of cytochrome oxidases consuming oxygen. Biochim Biophys Acta Bioenerg. 2020:1861(12):148304. 10.1016/j.bbabio.2020.148304 32890468

[koaf101-B28] Fernandez-Pozo N, Haas FB, Meyberg R, Ullrich KK, Hiss M, Perroud P-F, Hanke S, Kratz V, Powell AF, Vesty EF, et al PEATmoss (Physcomitrella Expression Atlas Tool): a unified gene expression atlas for the model plant *Physcomitrella patens*. Plant J. 2020:102(1):165–177. 10.1111/tpj.14607 31714620

[koaf101-B29] Fernie AR, Cavalcanti JHF, Nunes-Nesi A. Metabolic roles of plant mitochondrial carriers. Biomolecules. 2020:10(7):1013. 10.3390/biom10071013 32650612 PMC7408384

[koaf101-B30] Florez-Sarasa I, Welchen E, Racca S, Gonzalez DH, Vallarino JG, Fernie AR, Ribas-Carbo M, Del-Saz NF. Cytochrome c deficiency differentially affects the in vivo mitochondrial electron partitioning and primary metabolism depending on the photoperiod. Plants. 2021:10(3):444. 10.3390/plants10030444 33652808 PMC7996904

[koaf101-B31] Forde BG, Lea PJ. Glutamate in plants: metabolism, regulation, and signalling. J Exp Bot. 2007:58(9):2339–2358. 10.1093/jxb/erm121 17578865

[koaf101-B32] Forsum O, Svennerstam H, Ganeteg U, Näsholm T. Capacities and constraints of amino acid utilization in Arabidopsis. New Phytol. 2008:179(4):1058–1069. 10.1111/j.1469-8137.2008.02546.x 18627491

[koaf101-B33] Fricaud A-C, Walters AJ, Whitehouse DG, Moore AL. The role(s) of adenylate kinase and the adenylate carrier in the regulation of plant mitochondrial respiratory activity. Biochim Biophys Acta Bioenerg. 1992:1099(3):253–261. 10.1016/0005-2728(92)90035-Z

[koaf101-B34] Fromm S, Braun H-P, Peterhansel C. Mitochondrial gamma carbonic anhydrases are required for Complex I assembly and plant reproductive development. New Phytol. 2016a:211(1):194–207. 10.1111/nph.13886 26889912

[koaf101-B35] Fromm S, Senkler J, Eubel H, Peterhänsel C, Braun H-P. Life without Complex I: proteome analyses of an Arabidopsis mutant lacking the mitochondrial NADH dehydrogenase complex. J Exp Bot. 2016b:67(10):3079–3093. 10.1093/jxb/erw165 27122571 PMC4867900

[koaf101-B36] Fu Y-F, Zhang Z-W, Yuan S. Putative connections between nitrate reductase S-nitrosylation and NO synthesis under pathogen attacks and abiotic stresses. Front Plant Sci. 2018:9:474. 10.3389/fpls.2018.00474 29696031 PMC5905236

[koaf101-B37] Gardeström P, Igamberdiev AU. The origin of cytosolic ATP in photosynthetic cells. Physiol Plant. 2016:157(3):367–379. 10.1111/ppl.12455 27087668

[koaf101-B38] Gardeström P, Igamberdiev AU, Raghavendra AS. Mitochondrial functions in the light and significance to carbon–nitrogen interactions. In: Foyer CH, Noctor G, editors. Photosynthetic nitrogen assimilation and associated carbon and respiratory metabolism. Dordrecht: Springer Netherlands; 2002. p. 151–172.

[koaf101-B39] Gaufichon L, Reisdorf-Cren M, Rothstein SJ, Chardon F, Suzuki A. Biological functions of asparagine synthetase in plants. Plant Sci. 2010:179(3):141–153. 10.1016/j.plantsci.2010.04.010

[koaf101-B40] Gauthier PPG, Bligny R, Gout E, Mahé A, Nogués S, Hodges M, Tcherkez GGB. In folio isotopic tracing demonstrates that nitrogen assimilation into glutamate is mostly independent from current CO_2_ assimilation in illuminated leaves of *Brassica napus*. New Phytol. 2010:185(4):988–999. 10.1111/j.1469-8137.2009.03130.x 20070539

[koaf101-B41] Ge SX, Son EW, Yao R. iDEP: an integrated web application for differential expression and pathway analysis of RNA-seq data. BMC Bioinformatics. 2018:19(1):534. 10.1186/s12859-018-2486-6 30567491 PMC6299935

[koaf101-B42] Geigenberger P, Fernie AR. Metabolic control of redox and redox control of metabolism in plants. Antioxid Redox Signal. 2014:21(9):1389–1421. 10.1089/ars.2014.6018 24960279 PMC4158967

[koaf101-B43] Giraud E, Van Aken O, Ho LHM, Whelan J. The transcription factor ABI4 is a regulator of mitochondrial retrograde expression of ALTERNATIVE OXIDASE1a. Plant Physiol. 2009:150(3):1286–1296. 10.1104/pp.109.139782 19482916 PMC2705018

[koaf101-B44] Gout E, Rébeillé F, Douce R, Bligny R. Interplay of Mg2+, ADP, and ATP in the cytosol and mitochondria: unravelling the role of Mg2+ in cell respiration. Proc Natl Acad Sci U S A. 2014:111(43):E4560–E4567. 10.1073/pnas.1406251111 25313036 PMC4217410

[koaf101-B45] Gras DE, Mansilla N, Rodríguez C, Welchen E, Gonzalez DH. *Arabidopsis thaliana* SURFEIT1-like genes link mitochondrial function to early plant development and hormonal growth responses. Plant J. 2020:103(2):690–704. 10.1111/tpj.14762 32248588

[koaf101-B46] Gutierres S, Sabar M, Lelandais C, Chetrit P, Diolez P, Degand H, Boutry M, Vedel F, de Kouchkovsky Y, De Paepe R. Lack of mitochondrial and nuclear-encoded subunits of Complex I and alteration of the respiratory chain in *Nicotiana sylvestris* mitochondrial deletion mutants. Proc Natl Acad Sci U S A. 1997:94(7):3436–3441. 10.1073/pnas.94.7.3436 9096412 PMC20388

[koaf101-B47] Hager J, Pellny TK, Mauve C, Lelarge-Trouverie C, de Paepe R, Foyer CH, Noctor G. Conditional modulation of NAD levels and metabolite profiles in *Nicotiana sylvestris* by mitochondrial electron transport and carbon/nitrogen supply. Planta. 2010:231(5):1145–1157. 10.1007/s00425-010-1117-x 20182741

[koaf101-B48] Hampp R, Goller M, Ziegler H. Adenylate levels, energy charge, and phosphorylation potential during dark–light and light–dark transition in chloroplasts, mitochondria, and cytosol of mesophyll protoplasts from *Avena sativa* L. Plant Physiol. 1982:69(2):448–455. 10.1104/pp.69.2.448 16662227 PMC426228

[koaf101-B49] Hawkins C, Ginzburg D, Zhao K, Dwyer W, Xue B, Xu A, Rice S, Cole B, Paley S, Karp P, et al Plant metabolic network 15: a resource of genome-wide metabolism databases for 126 plants and algae. J Integr Plant Biol. 2021:63(11):1888–1905. 10.1111/jipb.13163 34403192

[koaf101-B50] Heber U, Santarius KA. Direct and indirect transfer of ATP and ADP across the chloroplast envelope. Zeitschrift für Naturforschung B. 1970:25(7):718–728. 10.1515/znb-1970-0714 4394211

[koaf101-B51] Higashi Y, Takechi K, Takano H, Takio S. Involvement of microRNA in copper deficiency-induced repression of chloroplastic CuZn-superoxide dismutase genes in the moss *Physcomitrella patens*. Plant Cell Physiol. 2013:54(8):1345–1355. 10.1093/pcp/pct084 23749811

[koaf101-B52] Hodges M . Enzyme redundancy and the importance of 2-oxoglutarate in plant ammonium assimilation. J Exp Bot. 2002:53(370):905–916. 10.1093/jexbot/53.370.905 11912233

[koaf101-B53] Igamberdiev AU . Citrate valve integrates mitochondria into photosynthetic metabolism. Mitochondrion. 2020:52:218–230. 10.1016/j.mito.2020.04.003 32278088

[koaf101-B54] Igamberdiev AU, Bykova NV. Mitochondria in photosynthetic cells: coordinating redox control and energy balance. Plant Physiol. 2023:191(4):2104–2119. 10.1093/plphys/kiac541 36440979 PMC10069911

[koaf101-B55] Ishikawa M, Morishita M, Higuchi Y, Ichikawa S, Ishikawa T, Nishiyama T, Kabeya Y, Hiwatashi Y, Kurata T, Kubo M, et al Physcomitrella STEMIN transcription factor induces stem cell formation with epigenetic reprogramming. Nat Plants. 2019:5(7):681–690. 10.1038/s41477-019-0464-2 31285563

[koaf101-B56] Järvi S, Suorsa M, Paakkarinen V, Aro E-M. Optimized native gel systems for separation of thylakoid protein complexes: novel super- and mega-complexes. Biochemical J. 2011:439(2):207–214. 10.1042/BJ20102155 21707535

[koaf101-B57] Kabbage M, Dickman MB. The BAG proteins: a ubiquitous family of chaperone regulators. Cell Mol Life Sci. 2008:65(9):1390–1402. 10.1007/s00018-008-7535-2 18264803 PMC11131705

[koaf101-B58] Karp PD, Midford PE, Billington R, Kothari A, Krummenacker M, Latendresse M, Ong WK, Subhraveti P, Caspi R, Fulcher C, et al Pathway tools version 23.0 update: software for pathway/genome informatics and systems biology. Brief Bioinform. 2021:22(1):109–126. 10.1093/bib/bbz104 31813964 PMC8453236

[koaf101-B59] Kaye Y, Huang W, Clowez S, Saroussi S, Idoine A, Sanz-Luque E, Grossman AR. The mitochondrial alternative oxidase from *Chlamydomonas reinhardtii* enables survival in high light. J Biol Chem. 2019:294(4):1380–1395. 10.1074/jbc.RA118.004667 30510139 PMC6349123

[koaf101-B60] Kolli R, Soll J, Carrie C. OXA2b is crucial for proper membrane insertion of COX2 during biogenesis of Complex IV in plant mitochondria. Plant Physiol. 2019:179(2):601–615. 10.1104/pp.18.01286 30487140 PMC6426407

[koaf101-B61] Krämer M, Kunz H-H. Indirect export of reducing equivalents from the chloroplast to resupply NADP for C3 photosynthesis—growing importance for stromal NAD(H)? Front Plant Sci. 2021:12:719003. 10.3389/fpls.2021.719003 34745158 PMC8564385

[koaf101-B62] Kromer S . Respiration during photosynthesis. Annu Rev Plant Biol. 1995:46(1):45–70. 10.1146/annurev.pp.46.060195.000401

[koaf101-B63] Lang D, Ullrich KK, Murat F, Fuchs J, Jenkins J, Haas FB, Piednoel M, Gundlach H, Van Bel M, Meyberg R, et al The *Physcomitrella patens* chromosome-scale assembly reveals moss genome structure and evolution. Plant J. 2018:93(3):515–533. 10.1111/tpj.13801 29237241

[koaf101-B64] Lange PR, Geserick C, Tischendorf G, Zrenner R. Functions of chloroplastic adenylate kinases in Arabidopsis. Plant Physiol. 2008:146(2):492–504. 10.1104/pp.107.114702 18162585 PMC2245825

[koaf101-B65] Langmead B, Salzberg SL. Fast gapped-read alignment with Bowtie 2. Nat Methods. 2012:9(4):357–359. 10.1038/nmeth.1923 22388286 PMC3322381

[koaf101-B66] Larosa V, Meneghesso A, La Rocca N, Steinbeck J, Hippler M, Szabò I, Morosinotto T. Mitochondria affect photosynthetic electron transport and photo-sensitivity in a green alga. Plant Physiol. 2018:176(3):2305–2314. 10.1104/pp.17.01249 29284743 PMC5841685

[koaf101-B67] Le Gall H, Philippe F, Domon J-M, Gillet F, Pelloux J, Rayon C. Cell wall metabolism in response to abiotic stress. Plants. 2015:4(1):112–166. 10.3390/plants4010112 27135320 PMC4844334

[koaf101-B68] Liao H-S, Chung Y-H, Hsieh M-H. Glutamate: a multifunctional amino acid in plants. Plant Sci. 2022:318:111238. 10.1016/j.plantsci.2022.111238 35351313

[koaf101-B69] Liu Y-J, Han X-M, Ren L-L, Yang H-L, Zeng Q-Y. Functional divergence of the glutathione S-transferase supergene family in *Physcomitrella patens* reveals complex patterns of large gene family evolution in land plants. Plant Physiol. 2013:161(2):773–786. 10.1104/pp.112.205815 23188805 PMC3561018

[koaf101-B70] Lloyd JPB, Lang D, Zimmer AD, Causier B, Reski R, Davies B. The loss of SMG1 causes defects in quality control pathways in *Physcomitrella patens*. Nucleic Acids Res. 2018:46(11):5822–5836. 10.1093/nar/gky225 29596649 PMC6009662

[koaf101-B71] Mackenzie S, McIntosh L. Higher plant mitochondria. Plant Cell. 1999:11(4):571–586. 10.1105/tpc.11.4.571 10213779 PMC144202

[koaf101-B72] Maeda H, Dudareva N. The shikimate pathway and aromatic amino acid biosynthesis in plants. Annu Rev Plant Biol. 2012:63(1):73–105. 10.1146/annurev-arplant-042811-105439 22554242

[koaf101-B73] Mansilla N, Garcia L, Gonzalez DH, Welchen E. AtCOX10, a protein involved in haem o synthesis during cytochrome c oxidase biogenesis, is essential for plant embryogenesis and modulates the progression of senescence. J Exp Bot. 2015:66(21):6761–6775. 10.1093/jxb/erv381 26246612

[koaf101-B74] Mellon M, Storti M, Vera-Vives AM, Kramer DM, Alboresi A, Morosinotto T. Inactivation of mitochondrial Complex I stimulates chloroplast ATPase in *Physcomitrium patens*. Plant Physiol. 2021:187(2):931–946. 10.1093/PLPHYS/KIAB276 34608952 PMC8491079

[koaf101-B75] Meyer EH, Tomaz T, Carroll AJ, Estavillo G, Delannoy E, Tanz SK, Small ID, Pogson BJ, Millar AH. Remodeled respiration in ndufs4 with low phosphorylation efficiency suppresses Arabidopsis germination and growth and alters control of metabolism at night. Plant Physiol. 2009:151(2):603–619. 10.1104/pp.109.141770 19675153 PMC2754622

[koaf101-B76] Meyer EH, Welchen E, Carrie C. Assembly of the complexes of the oxidative phosphorylation system in land plant mitochondria. Annu Rev Plant Biol. 2019:70(1):23–50. 10.1146/annurev-arplant-050718-100412 30822116

[koaf101-B77] Millar AH, Whelan J, Soole KL, Day DA. Organization and regulation of mitochondrial respiration in plants. Annu Rev Plant Biol. 2011:62(1):79–104. 10.1146/annurev-arplant-042110-103857 21332361

[koaf101-B78] Moreno-García B, López-Calcagno PE, Raines CA, Sweetlove LJ. Suppression of metabolite shuttles for export of chloroplast and mitochondrial ATP and NADPH increases the cytosolic NADH:NAD+ ratio in tobacco leaves in the dark. J Plant Physiol. 2022:268:153578. 10.1016/j.jplph.2021.153578 34911031

[koaf101-B79] Noctor G, De Paepe R, Foyer CH. Mitochondrial redox biology and homeostasis in plants. Trends Plant Sci. 2007:12(3):125–134. 10.1016/j.tplants.2007.01.005 17293156

[koaf101-B80] Noguchi K, Yoshida K. Interaction between photosynthesis and respiration in illuminated leaves. Mitochondrion. 2008:8(1):87–99. 10.1016/j.mito.2007.09.003 18024239

[koaf101-B81] Pellny TK, Van Aken O, Dutilleul C, Wolff T, Groten K, Bor M, De Paepe R, Reyss A, Van Breusegem F, Noctor G, et al Mitochondrial respiratory pathways modulate nitrate sensing and nitrogen-dependent regulation of plant architecture in *Nicotiana sylvestris*. Plant J. 2008:54(6):976–992. 10.1111/j.1365-313X.2008.03472.x 18318685 PMC2440565

[koaf101-B82] Peters K, Nießen M, Peterhänsel C, Späth B, Hölzle A, Binder S, Marchfelder A, Braun H-P. Complex I–Complex II ratio strongly differs in various organs of *Arabidopsis thaliana*. Plant Mol Biol. 2012:79(3):273–284. 10.1007/s11103-012-9911-4 22527752

[koaf101-B83] Pétriacq P, de Bont L, Genestout L, Hao J, Laureau C, Florez-Sarasa I, Rzigui T, Queval G, Gilard F, Mauve C, et al Photoperiod affects the phenotype of mitochondrial Complex I mutants. Plant Physiol. 2017:173(1):434–455. 10.1104/pp.16.01484 27852950 PMC5210746

[koaf101-B84] Pfister B, Zeeman SC. Formation of starch in plant cells. Cell Mol Life Sci. 2016:73(14):2781–2807. 10.1007/s00018-016-2250-x 27166931 PMC4919380

[koaf101-B85] Pineau B, Layoune O, Danon A, De Paepe R. L-Galactono-1,4-lactone dehydrogenase is required for the accumulation of plant respiratory Complex I. J Biol Chem. 2008:283(47):32500–32505. 10.1074/jbc.M805320200 18799460

[koaf101-B86] Porra RJ, Thompson WA, Kriedemann PE. Determination of accurate extinction coefficients and simultaneous equations for assaying chlorophylls a and b extracted with four different solvents: verification of the concentration of chlorophyll standards by atomic absorption spectroscopy. Biochim Biophys Acta Bioenerg. 1989:975(3):384–394. 10.1016/S0005-2728(89)80347-0

[koaf101-B87] Pu X, Yang L, Liu L, Dong X, Chen S, Chen Z, Liu G, Jia Y, Yuan W, Liu L. Genome-wide analysis of the MYB transcription factor superfamily in *Physcomitrella patens*. Int J Mol Sci. 2020:21(3):975. 10.3390/ijms21030975 32024128 PMC7037163

[koaf101-B88] Putri GH, Anders S, Pyl PT, Pimanda JE, Zanini F. Analysing high-throughput sequencing data in Python with HTSeq 2.0. Bioinformatics. 2022:38(10):2943–2945. 10.1093/bioinformatics/btac166 35561197 PMC9113351

[koaf101-B89] Racca S, Welchen E, Gras DE, Tarkowská D, Turečková V, Maurino VG, Gonzalez DH. Interplay between cytochrome c and gibberellins during Arabidopsis vegetative development. Plant J. 2018:94(1):105–121. 10.1111/tpj.13845 29385297

[koaf101-B90] Radin I, Mansilla N, Rödel G, Steinebrunner I. The Arabidopsis COX11 homolog is essential for cytochrome c oxidase activity. Front Plant Sci. 2015:6:1091. 10.3389/fpls.2015.01091 26734017 PMC4683207

[koaf101-B91] Ritchie ME, Phipson B, Wu D, Hu Y, Law CW, Shi W, Smyth GK. Limma powers differential expression analyses for RNA-sequencing and microarray studies. Nucleic Acids Res. 2015:43(7):e47. 10.1093/nar/gkv007 25605792 PMC4402510

[koaf101-B92] Sabar M, De Paepe R, de Kouchkovsky Y. Complex I impairment, respiratory compensations, and photosynthetic decrease in nuclear and mitochondrial male sterile mutants of *Nicotiana sylvestris* 1. Plant Physiol. 2000:124(3):1239–1250. 10.1104/pp.124.3.1239 11080300 PMC59222

[koaf101-B93] Sakakibara K, Nishiyama T, Sumikawa N, Kofuji R, Murata T, Hasebe M. Involvement of auxin and a homeodomain-leucine zipper I gene in rhizoid development of the moss *Physcomitrella patens*. Development (Cambridge, England). 2003:130(20):4835–4846. 10.1242/dev.00644 12917289

[koaf101-B94] Salinas T, Larosa V, Cardol P, Maréchal-Drouard L, Remacle C. Respiratory-deficient mutants of the unicellular green alga *Chlamydomonas*: a review. Biochimie. 2014:100(1):207–218. 10.1016/j.biochi.2013.10.006 24139906

[koaf101-B95] Schindelin J, Arganda-Carreras I, Frise E, Kaynig V, Longair M, Pietzsch T, Preibisch S, Rueden C, Saalfeld S, Schmid B, et al Fiji: an open-source platform for biological-image analysis. Nat Methods. 2012:9(7):676–682. 10.1038/nmeth.2019 22743772 PMC3855844

[koaf101-B96] Shameer S, Ratcliffe RG, Sweetlove LJ. Leaf energy balance requires mitochondrial respiration and export of chloroplast NADPH in the light. Plant Physiol. 2019:180(4):1947–1961. 10.1104/pp.19.00624 31213510 PMC6670072

[koaf101-B97] Shikanai T, Yamamoto H. Contribution of cyclic and pseudo-cyclic electron transport to the formation of proton motive force in chloroplasts. Mol Plant. 2017:10(1):20–29. 10.1016/j.molp.2016.08.004 27575692

[koaf101-B98] Shim S-H, Lee S-K, Lee D-W, Brilhaus D, Wu G, Ko S, Lee C-H, Weber APM, Jeon J-S. Loss of function of rice plastidic glycolate/glycerate translocator 1 impairs photorespiration and plant growth. Front Plant Sci. 2020:10:1726. 10.3389/fpls.2019.01726 32038690 PMC6993116

[koaf101-B99] Sievers F, Wilm A, Dineen D, Gibson TJ, Karplus K, Li W, Lopez R, McWilliam H, Remmert M, Söding J, et al Fast, scalable generation of high-quality protein multiple sequence alignments using Clustal omega. Mol Syst Biol. 2011:7(1):539. 10.1038/msb.2011.75 21988835 PMC3261699

[koaf101-B100] Smith AM, Zeeman SC. Quantification of starch in plant tissues. Nat Protoc. 2006:1(3):1342–1345. 10.1038/nprot.2006.232 17406420

[koaf101-B101] Steinebrunner I, Landschreiber M, Krause-Buchholz U, Teichmann J, Rödel G. HCC1, the Arabidopsis homologue of the yeast mitochondrial copper chaperone SCO1, is essential for embryonic development. J Exp Bot. 2011:62(1):319–330. 10.1093/jxb/erq269 21041373

[koaf101-B102] Stitt M, Zeeman SC. Starch turnover: pathways, regulation and role in growth. Curr Opin Plant Biol. 2012:15(3):282–292. 10.1016/j.pbi.2012.03.016 22541711

[koaf101-B103] Stocking CR, Larson S. A chloroplast cytoplasmic shuttle and the reduction of extraplastid nad. Biochem Biophys Res Commun. 1969:37(2):278–282. 10.1016/0006-291X(69)90731-1 4390456

[koaf101-B104] Subrahmanian N, Remacle C, Hamel PP. Plant mitochondrial Complex I composition and assembly: a review. Biochim Biophys Acta Bioenerg. 2016:1857(7):1001–1014. 10.1016/j.bbabio.2016.01.009 26801215

[koaf101-B105] Suzuki N, Koussevitzky S, Mittler R, Miller G. ROS and redox signalling in the response of plants to abiotic stress. Plant Cell Environ. 2012:35(2):259–270. 10.1111/j.1365-3040.2011.02336.x 21486305

[koaf101-B106] Sweetlove LJ, Beard KFM, Nunes-Nesi A, Fernie AR, Ratcliffe RG. Not just a circle: flux modes in the plant TCA cycle. Trends Plant Sci. 2010:15(8):462–470. 10.1016/j.tplants.2010.05.006 20554469

[koaf101-B107] Szal B, Podgórska A. The role of mitochondria in leaf nitrogen metabolism. Plant Cell Environ. 2012:35(10):1756–1768. 10.1111/j.1365-3040.2012.02559.x 22697909

[koaf101-B108] Tang D, Chen M, Huang X, Zhang G, Zeng L, Zhang G, Wu S, Wang Y. SRplot: a free online platform for data visualization and graphing. PLoS One. 2023:18(11):e0294236. 10.1371/journal.pone.0294236 37943830 PMC10635526

[koaf101-B109] Tcherkez G, Gauthier P, Buckley TN, Busch FA, Barbour MM, Bruhn D, Heskel MA, Gong XY, Crous KY, Griffin K, et al Leaf day respiration: low CO_2_ flux but high significance for metabolism and carbon balance. New Phytol. 2017:216(4):986–1001. 10.1111/nph.14816 28967668

[koaf101-B110] Terasawa K, Odahara M, Kabeya Y, Kikugawa T, Sekine Y, Fujiwara M, Sato N. The mitochondrial genome of the moss *Physcomitrella patens* sheds new light on mitochondrial evolution in land plants. Mol Biol Evol. 2007:24(3):699–709. 10.1093/molbev/msl198 17175527

[koaf101-B111] Timón-Gómez A, Nývltová E, Abriata LA, Vila AJ, Hosler J, Barrientos A. Mitochondrial cytochrome *c* oxidase biogenesis: recent developments. Semin Cell Dev Biol. 2018:76:163–178. 10.1016/j.semcdb.2017.08.055 28870773 PMC5842095

[koaf101-B112] Tyutereva EV, Murtuzova AV, Voitsekhovskaja OV. Autophagy and the energy status of plant cells. Russ J Plant Physiol. 2022:69(2):19. 10.1134/S1021443722020212

[koaf101-B113] Van Aken O . Mitochondrial redox systems as central hubs in plant metabolism and signaling. Plant Physiol. 2021:186(1):36–52. 10.1093/plphys/kiab101 33624829 PMC8154082

[koaf101-B114] Vandeleur RK, Sullivan W, Athman A, Jordans C, Gilliham M, Kaiser BN, Tyerman SD. Rapid shoot-to-root signalling regulates root hydraulic conductance via aquaporins. Plant Cell Environ. 2014:37(2):520–538. 10.1111/pce.12175 23926961

[koaf101-B115] Vanlerberghe GC, Dahal K, Alber NA, Chadee A. Photosynthesis, respiration and growth: a carbon and energy balancing act for alternative oxidase. Mitochondrion. 2020:52:197–211. 10.1016/j.mito.2020.04.001 32278748

[koaf101-B116] Vera-Vives AM, Novel P, Zheng K, Tan S-L, Schwarzländer M, Alboresi A, Morosinotto T. Mitochondrial respiration is essential for photosynthesis-dependent ATP supply of the plant cytosol. New Phytol. 2024:243(6):2175–2186. 10.1111/nph.19989 39073122

[koaf101-B117] Vidal G, Ribas-Carbo M, Garmier M, Dubertret G, Rasmusson AG, Mathieu C, Foyer CH, De Paepe R. Lack of respiratory chain Complex I impairs alternative oxidase engagement and modulates redox signaling during elicitor-induced cell death in tobacco. Plant Cell. 2007:19(2):640–655. 10.1105/tpc.106.044461 17277035 PMC1867325

[koaf101-B118] Voon CP, Law Y-S, Guan X, Lim S-L, Xu Z, Chu W-T, Zhang R, Sun F, Labs M, Leister D, et al Modulating the activities of chloroplasts and mitochondria promotes adenosine triphosphate production and plant growth. Quant Plant Biol. 2021:2:e7. 10.1017/qpb.2021.7 37077204 PMC10095973

[koaf101-B119] Waters ER, Vierling E. Plant small heat shock proteins—evolutionary and functional diversity. New Phytol. 2020:227(1):24–37. 10.1111/nph.16536 32297991

[koaf101-B120] Went N, Di Marcello M, Gnaiger E. Oxygen dependence of photosynthesis and light-enhanced dark respiration studied by high-resolution PhotoRespirometry. MitoFit preprint. 10.26124/mitofit:2021-0005, 2021, preprint: not peer reviewed.

[koaf101-B121] Winter G, Todd CD, Trovato M, Forlani G, Funck D. Physiological implications of arginine metabolism in plants. Front Plant Sci. 2015:6:534. 10.3389/fpls.2015.00534 26284079 PMC4520006

[koaf101-B122] Xia J, Wishart DS. Web-based inference of biological patterns, functions and pathways from metabolomic data using MetaboAnalyst. Nat Protoc. 2011:6(6):743–760. 10.1038/nprot.2011.319 21637195

[koaf101-B123] Xu L, Carrie C, Law SR, Murcha MW, Whelan J. Acquisition, conservation, and loss of dual-targeted proteins in land plants. Plant Physiol. 2013:161(2):644–662. 10.1104/pp.112.210997 23257241 PMC3561010

[koaf101-B124] Yoneyama T, Suzuki A. Light-independent nitrogen assimilation in plant leaves: nitrate incorporation into glutamine, glutamate, aspartate, and asparagine traced by 15N. Plants. 2020:9(10):1303. 10.3390/plants9101303 33023108 PMC7600499

[koaf101-B125] Yoshida K, Terashima I, Noguchi K. Distinct roles of the cytochrome pathway and alternative oxidase in leaf photosynthesis. Plant Cell Physiol. 2006:47(1):22–31. 10.1093/pcp/pci219 16239307

[koaf101-B126] Zancani M, Braidot E, Filippi A, Lippe G. Structural and functional properties of plant mitochondrial F-ATP synthase. Mitochondrion. 2020:53:178–193. 10.1016/j.mito.2020.06.001 32534049

[koaf101-B127] Zhang J, Fu X-X, Li R-Q, Zhao X, Liu Y, Li M-H, Zwaenepoel A, Ma H, Goffinet B, Guan Y-L, et al The hornwort genome and early land plant evolution. Nat Plants. 2020:6(2):107–118. 10.1038/s41477-019-0588-4 32042158 PMC7027989

[koaf101-B128] Zhang Y, Swart C, Alseekh S, Scossa F, Jiang L, Obata T, Graf A, Fernie AR. The extra-pathway interactome of the TCA cycle: expected and unexpected metabolic interactions. Plant Physiol. 2018:177(3):966–979. 10.1104/pp.17.01687 29794018 PMC6052981

