# Supplementary material for: Cytochrome c oxidase inactivation in Physcomitrium patens reveals that respiration coordinates plant metabolism
Source: Plant Cell. 2025 May 5;37(6):koaf101. doi: 10.1093/plcell/koaf101 (PMC12164586; doi:10.1093/plcell/koaf101)
Supplement: koaf101_Supplementary_Data [file koaf101_supplementary_data.zip › Vera-Vives et al Supplementary material revised-ed.pdf]

Supplementary material

```
P.patens        MMNRWRCVKSLSEALHRHGLAAAASNSFTADLTLAKPLPNIYAKEASTSHSEKLVLPSVA 60
B.taurus        ------------------------------------------------------------ 0
S.cerevisiae    -----------------------------------------------------MIRICPI 7


P.patens        VRTDVHSLC--------------FLWSTLSRLSLNREYSTLLANHRFCSIPSRGGAEI-- 104
B.taurus        -------MGGLWRPAWRRVVFCGWSWSHLGRPT------RAAERAEPCLRPGRSGPAGTE 47
S.cerevisiae    VRSKVPLLGTFLRS---------DSWLA---PH------ALALRRAICKNVA-------- 41
                       :                 *                 .   *   .

P.patens        YGFRTRGFSSQ---AEIAAKA----------GARRAKLTWESQTAGKKKSEAMLMYLVAM 151
B.taurus        QGLRRLGTWRRPSPAEQPARR----------PKSTNPYTRSQEEDWRRRNKTVLTYMAAA 97
S.cerevisiae    --LRS---YS--VNSEQPKHTFDISKLTRNEIQQLRELKRAR--ERKFKDRTVAFYFSSV 92
                  :*          :*   :                  .       : :..::  *: :

P.patens        VTAMVGITYAAVPLYRKFCQATGYGGTVQRKETVEEKIARHKGEEAESSRELVVQFNADV 211
B.taurus        AVGMLGASYAAVPLYRLYCQTTGLGGSAVAGHASD---QIENMVPVK-DRIIKITFNADV 153
S.cerevisiae    AVLFLGLAYAAVPLYRAICARTGFGGIPITDRRKF---TDDKLIPVDTEKRIRISFTSEV 149
                .. ::* :********  *  ** **     .        .:   .. .: : : *.::*

P.patens        ADGMPWKFTPCQREIRVRPGQSTLAFYTAENTSSVPITGVSTYNVTPMKAGLYFNKIQCF 271
B.taurus        HASLQWNFRPQQTEIYVVPGETALAFYKAKNPTDKPVIGISTYNVVPFEAGQYFNKIQCF 213
S.cerevisiae    SQILPWKFVPQQREVYVLPGETALAFYKAKNYSDKDIIGMATYSIAPGEAAQYFNKIQCF 209
                   : *:* * * *: * **:::****.*:* :.  : *::**.:.* :*. ********

P.patens        CFEEQRLLPGEKIDMPVFFFIDPEFATDPKMKGINSLILSYTFFKVEEAPQKETVASPQP 331
B.taurus        CFEEQRLNPQEEVDMPVFFYIDPEFAEDPRMVNVDLITLSYTFFEAKEGHTLPVPGYNSN 273
S.cerevisiae    CFEEQKLAAGEEIDMPVFFFIDPDFASDPAMRNIDDIILHYTFFRAHYGDGTAVSDSKKE 269
                *****:*   *::******:***:** ** * .:: : * ****... .    .    .

P.patens          A------------------------------  332
B.taurus          QQLSPASNL----------------------  282
S.cerevisiae      PEMNADEKAASLANAAILSPEVIDTRKDNSN  300
```

Supplementary Figure S1. Multiple sequence alignment of protein COX11 of *P. patens* and their homologs in yeast (*Saccharomyces cerevisiae*) and cattle (*Bos taurus*). The multiple sequence alignment was performed using the tool *Clustal Omega (1.2.4).* Letters with yellow background are the conserved Cys residues involved in copper binding as identified by Carr et al., 2002. Supports Figure 1.

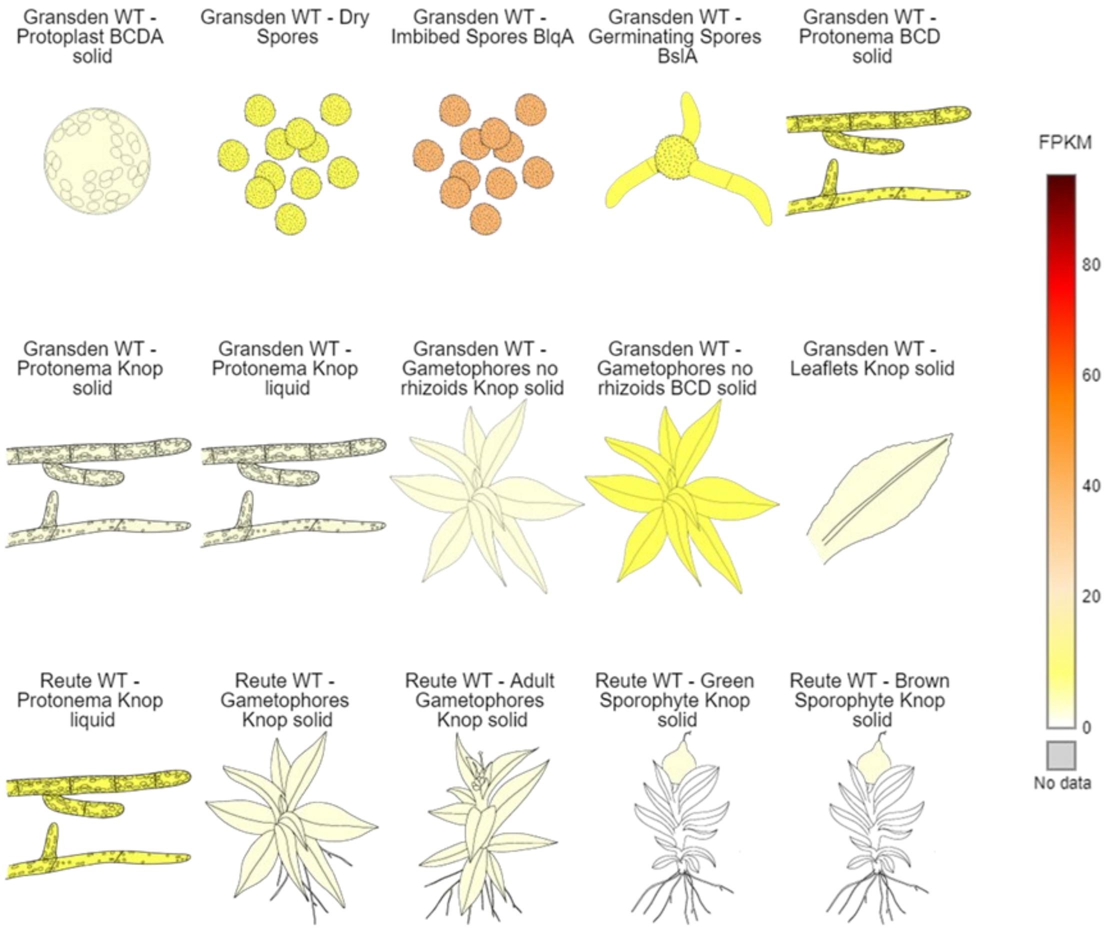

Supplementary Figure S2. **Expression levels of *COX11* in different developmental stages**. Note that the FPKM are relatively low for all stages, with imbibed spores presenting the highest expression. Data and figure were retrieved from the PeatMOSS Gene Atlas Database (Fernandez-Pozo et al., 2020). Supports Figure 1.

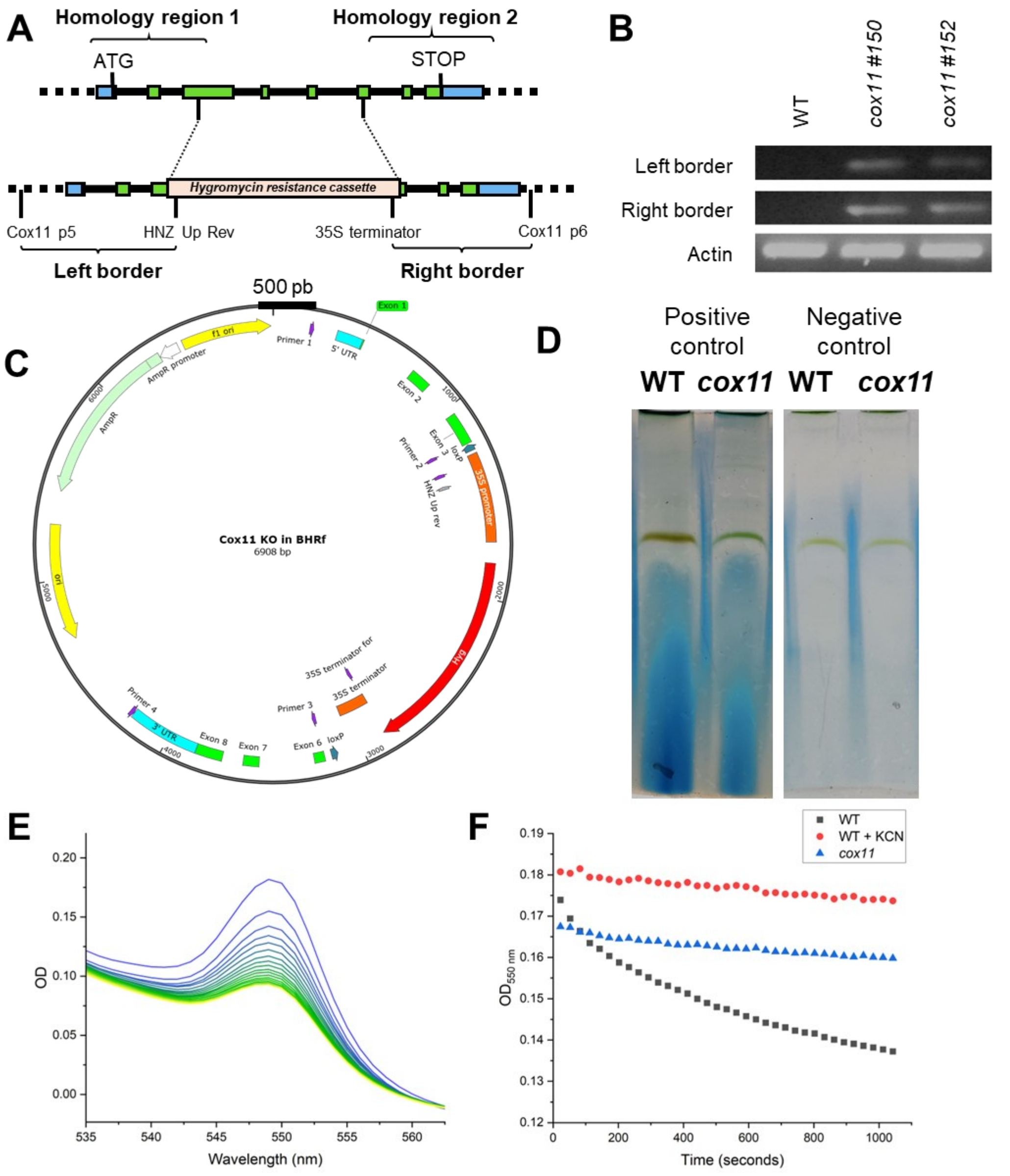

Supplementary Figure S3. *COX11* locus used for producing the knockout mutant lines and quantification of CIV activity. The gene map (A, top) and the corresponding region after construct insertion through homologous recombination (A, bottom) are shown. Green portions represent the exons, blue portions represent the 5’ and 3’ untranslated regions (UTR). The positions of the first and last codon of each coding region are marked with ATG and STOP, respectively. Integration of the antibiotic resistance cassette occurs through recombination of the respective homology regions. The two regions used for validation of their identity through PCR analysis using the given primers are marked as Left Border (LB) and Right Border (RB), and the corresponding bands are shown in B. Primer sequences are included in Supplementary table 1. C) Map of the construct used for knocking out *COX11*. Primer sequences are included in Supplementary Table 1. D) Example of In-gel activity staining after separation of crude membrane extracts by BN-PAGE with negative control of staining with 50 mM KCN. D) shows and example of spectroscopic quantification of CIV activity by incubation with reduced cyt c. the typical absorption peak at 550 nm decrease with the oxidation. Spectra with different colours (in a gradient going from blue to yellow) were taken every 30 seconds. E) Example of kinetics of OD_{550} for activity tests of WT, WT+KCN and *cox11*. Activities reported in figure 1 were quantified from the slope of the kinetics. Supports Figure 1.

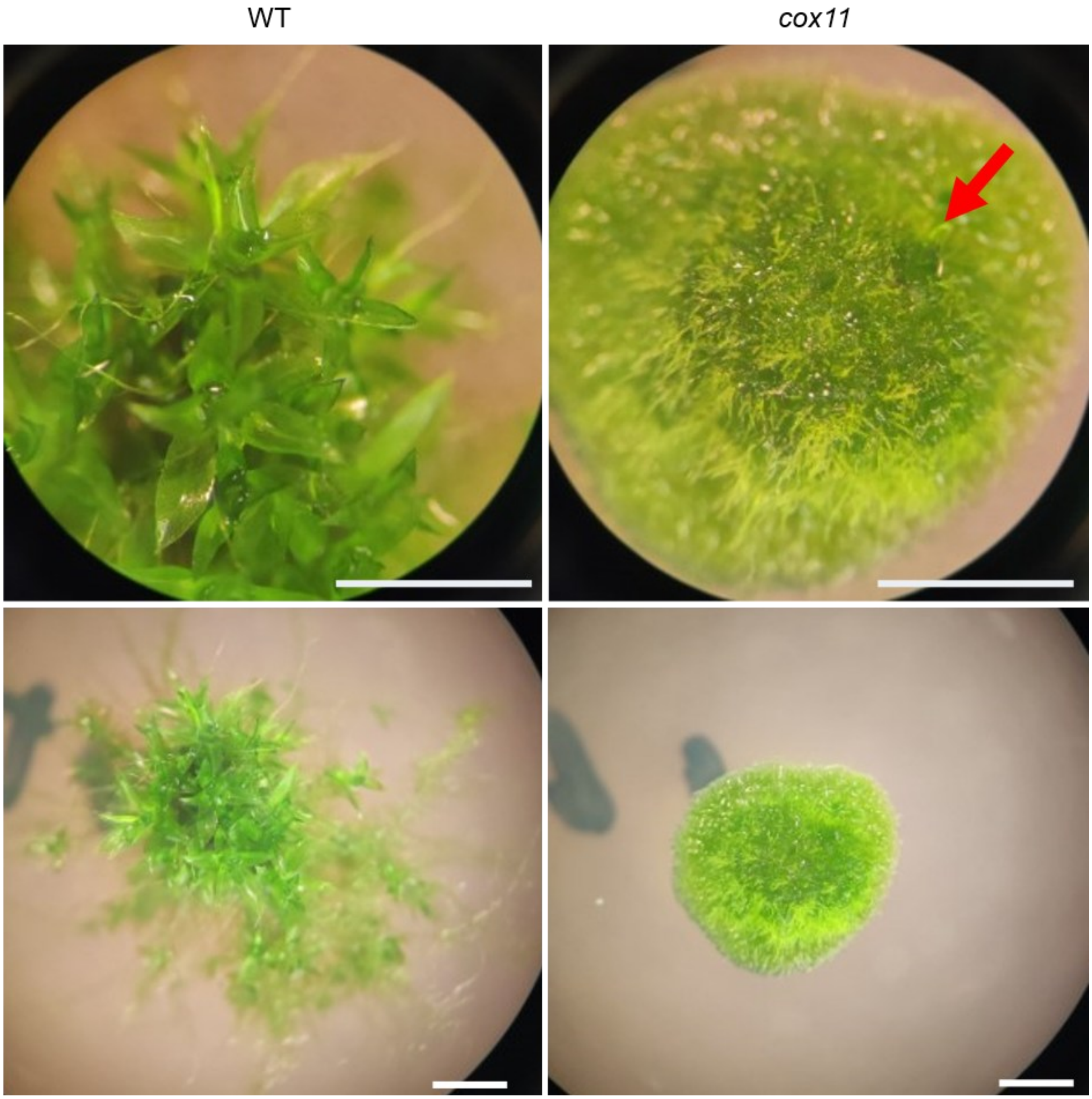

Supplementary Figure S4. Differential development of gametophores in *cox11*. Images of plant colonies after 21 days of growth, at two different magnifications. A developing gametophore is marked with a red arrow in *cox11*. Scale bars are 2 mm. Supports Figure 2.

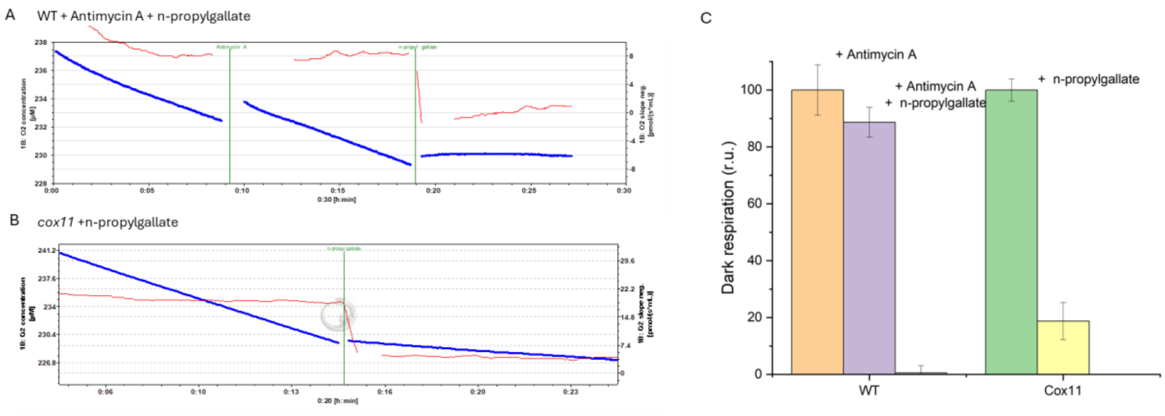

Supplementary Figure S5. Effect of antimycin and n-propylgallate on dark respiration of intact protonema. Two representative tracks of experiments of respirometry performed on WT (A) or *cox11* (B) are shown. The blue plot (left axis) shows O_2 concentration, while the red plot (right axis) shows O_2 flux. On WT, the addition of Antimycin A did not have an effect in O_2 consumption rate, but n-propylgallate abolished it (A). On *cox11*, n-propylgallate alone was sufficient to block all O_2 consumption (B). Images are screenshots of the DatLab software. C) Quantification of respiratory activity and impact of inhibitors (average ± SD, n > 4). Supports Figure 3.

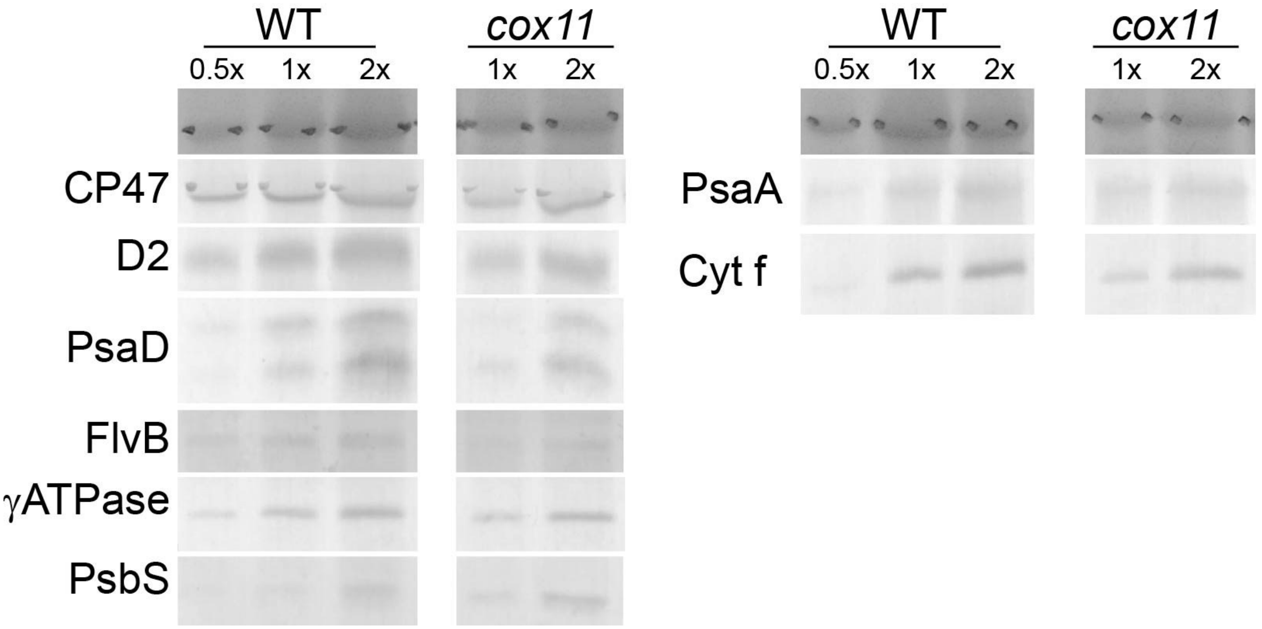

Supplementary Figure S6. Immunoblotting against subunits of the photosynthetic machinery. No major alterations in photosynthetic components were detected. 1× corresponds to 2 μg of loaded chlorophylls. Supports Figure 4.

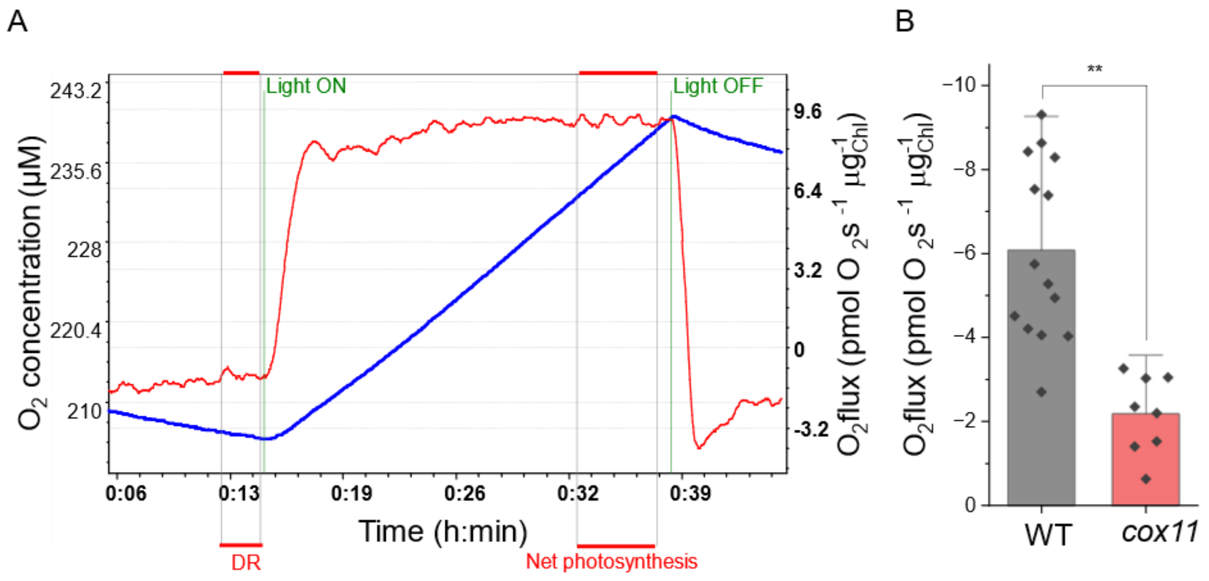

Supplementary Figure S7. Net photosynthesis measurement and values on intact protonema. (A) Representative track of one experiment of respirometry. The blue plot (left axis) shows O_2 concentration, while the red plot (right axis) shows O_2 flux. After quantification of dark respiration (DR), saturating light was turned on. When the O_2 flux was stable (red plot), the net photosynthesis was measured as the median of the shown stable region. (B) Values of net photosynthesis (±SD) from WT and *cox11*. Values in *cox11* were obtained from two independent lines. Statistics: (**) $p<0.01$, $n>8$. Supports Figure 4.

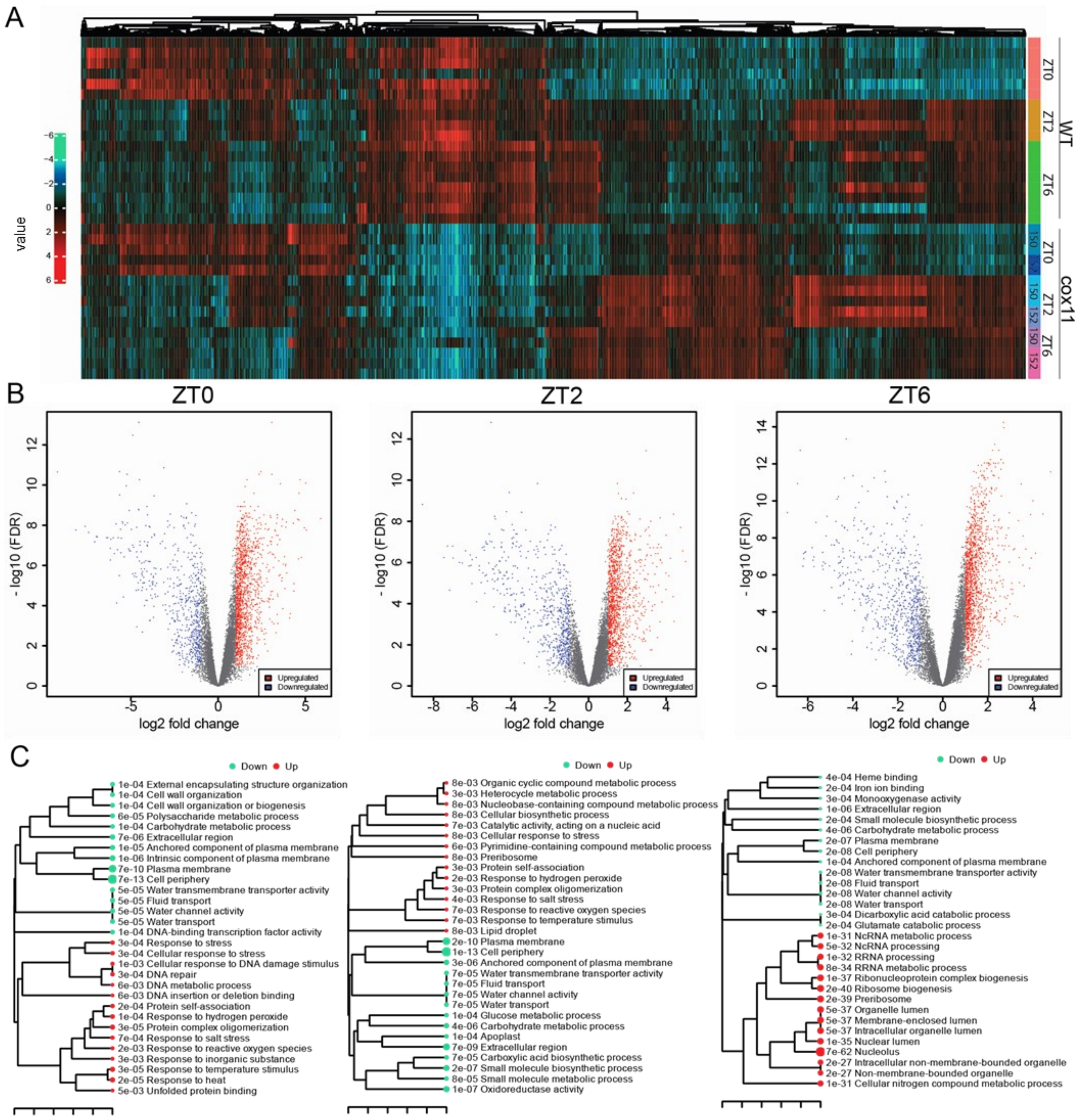

Supplementary Figure S8. RNA-seq data overview and enriched pathways segregated for the three zeitgeber times tested. (A) Hierarchical clustering of the 2,000 top genes, showing Pearson distance and average linkage. For each zeitgeber time, a volcano plot showing data distribution and the number of significant DEGs (B) and a tree of the most significant enriched pathways, where pathways with many shared genes are clustered together, and bigger dots indicate more significant p-values (C), are reported. Supports Figure 5.

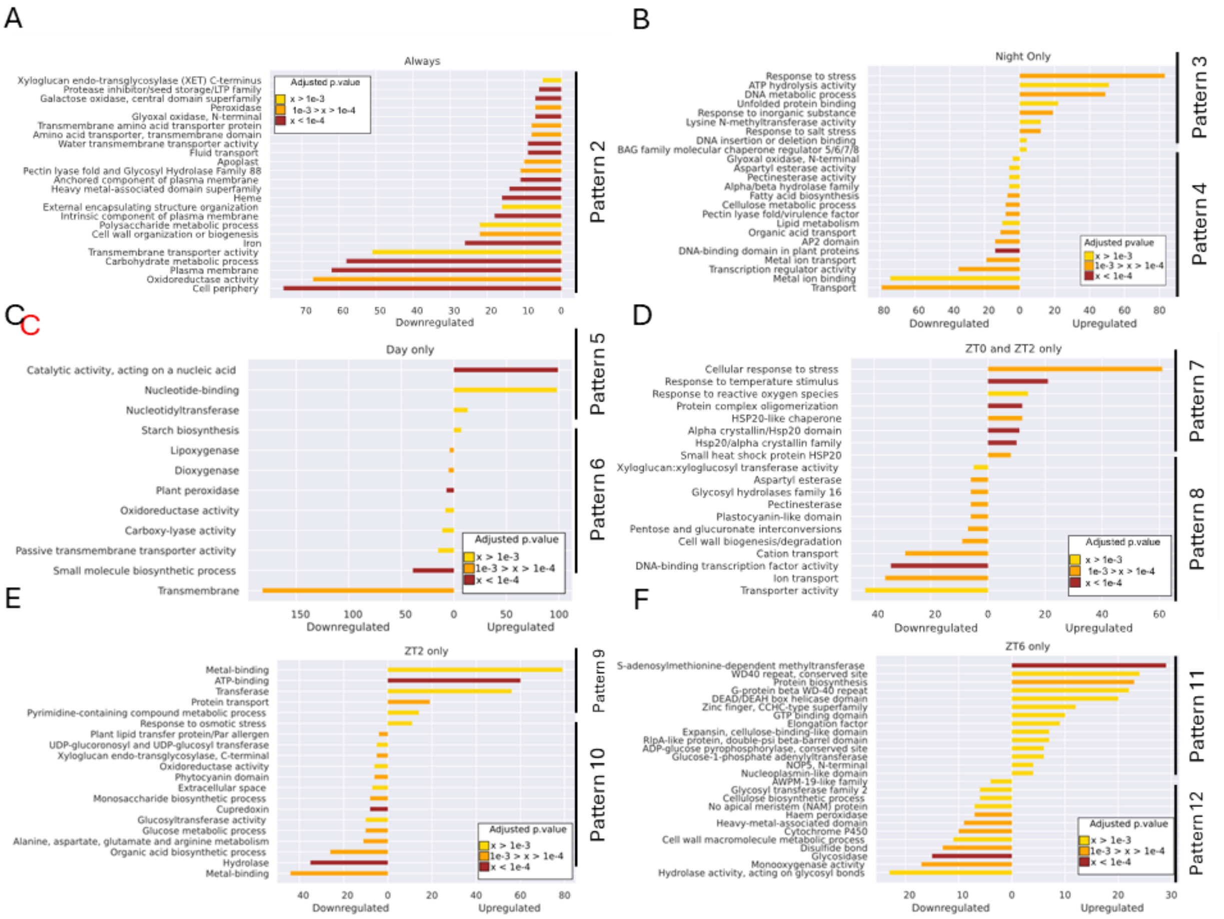

Supplementary Figure S9. Bar plots with the most significant pathways with differential expression in *cox11.* Different groups are distinguished following the pattern classification defined in Figure 5. Length of bars represents numbers of genes included, while colors represent adjusted p-values of the enriched pathway. Detailed information and composition of these pathways can be found in **Supplementary Dataset 2.** Supports Figure 5.

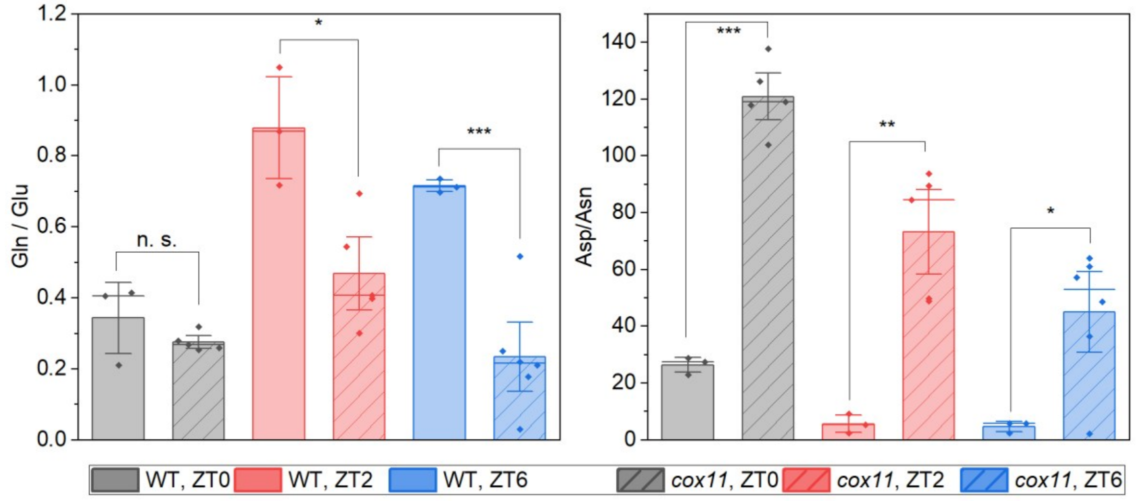

Supplementary Figure S10. **Ratios comparing relative abundances of glutamate/glutamine and aspartate/asparagine**. Statistics: average and SE are shown, two-sample t-test, (***) p<0.001; (**) p<0.01; (*) p>0.05; (n. s.) not significant, n =4.

A

WT

	Days							
	14	24	28	32	49	56	63	74
Ala 3 mM	Up	Up	Up	Up	Up	Up	Up	Up
Arg 3 mM	n. s.	Down	Up	n. s.	Up	Up	Up	Up
Asn 3 mM	Down	Up	Up	Up	Up	Up	Up	Up
Asp 3 mM	n. s.	Up	Up	Up	Up	Up	Up	Up
Cys 3 mM	Down	Down	Down	Down	Down	Down	n. s.	n. s.
Gln 3 mM	n. s.	Up	Up	Up	Up	Up	Up	Up
Glu 3 mM	n. s.	Down	n. s.	n. s.	n. s.	n. s.	n. s.	n. s.
Gly 3 mM	n. s.	Up	Up	Up	Up	Up	Up	Up
His 3 mM	Down	Down	Down	Down	Down	Down	Down	Down
Ile 3 mM	Down	Down	Down	Down	Down	Down	Down	Down
Leu 3 mM	Down	Down	Down	Down	Down	Down	Down	Down
Lys 3 mM	Down	Down	Down	Down	Down	Down	Down	Down
Met 3 mM	n. s.	Down	Down	Down	Down	Down	Down	Down
Phe 3 mM	Down	Down	Down	Down	Down	Down	Down	Down
Pro 3 mM	n. s.	Down	Down	Down	Down	Down	Down	Down
Ser 3 mM	n. s.	Up	Up	Up	Up	Up	Up	Up
Thr 3 mM	Down	Down	Down	Down	Down	Down	Down	Down
Trp 3 mM	Down	Down	Down	Down	Down	Down	Down	Down
Val 3 mM	Down	Down	Down	Down	Down	Down	Down	Down

cox11

	Days							
	14	24	28	32	49	56	63	74
Ala 3 mM	n. s.	n. s.	n. s.	n. s.	Up	Up	Up	Up
Arg 3 mM	n. s.	Down	n. s.	n. s.	n. s.	n. s.	Up	Up
Asn 3 mM	n. s.	n. s.	n. s.	n. s.	n. s.	n. s.	Up	Up
Asp 3 mM	n. s.	n. s.	n. s.	Up	Up	Up	Up	Up
Cys 3 mM	Down	Down	Down	Down	n. s.	n. s.	n. s.	n. s.
Gln 3 mM	n. s.	n. s.	Up	Up	Up	Up	Up	Up
Glu 3 mM	n. s.	n. s.	n. s.	n. s.	n. s.	n. s.	n. s.	n. s.
Gly 3 mM	Up	Up	Up	Up	Up	Up	Up	Up
His 3 mM	n. s.	Down	Down	Down	n. s.	n. s.	n. s.	n. s.
Ile 3 mM	n. s.	Down	Down	Down	n. s.	Down	n. s.	n. s.
Leu 3 mM	Down	Down	Down	Down	Down	Down	Down	Down
Lys 3 mM	n. s.	Down	Down	Down	n. s.	n. s.	n. s.	n. s.
Met 3 mM	n. s.	Down	Down	Down	n. s.	Down	n. s.	n. s.
Phe 3 mM	n. s.	Down	n. s.	n. s.	n. s.	n. s.	n. s.	n. s.
Pro 3 mM	n. s.	Down	Down	Down	n. s.	Down	n. s.	n. s.
Ser 3 mM	n. s.	Up	Up	Up	Up	Up	Up	Up
Thr 3 mM	n. s.	Down	Down	Down	Down	Down	Down	Down
Trp 3 mM	n. s.	Down	Down	Down	Down	Down	Down	Down
Val 3 mM	n. s.	Down	Down	Down	n. s.	Down	Down	Down

B

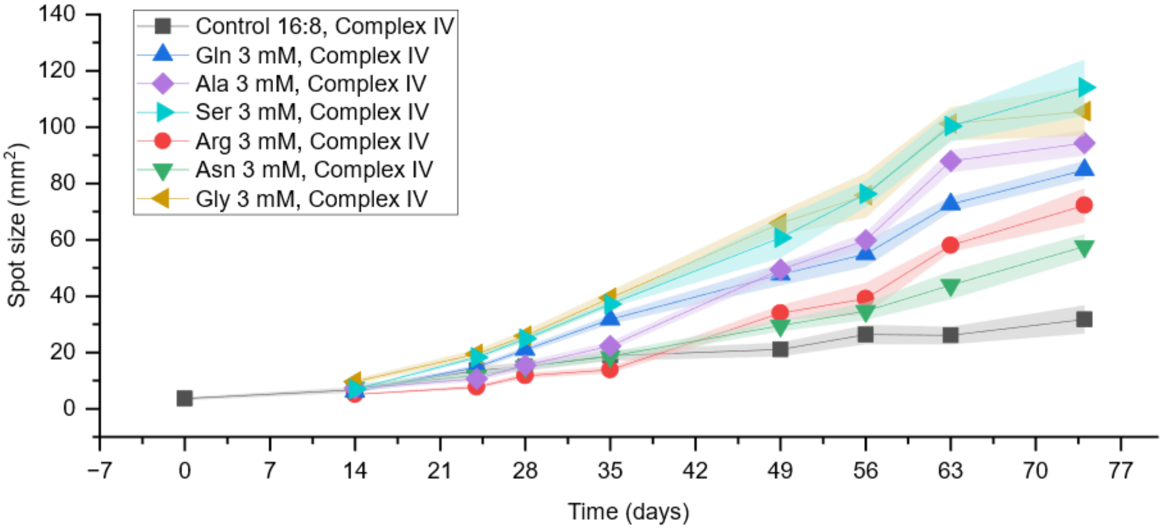

Supplementary Figure S11. **Effect of different amino acids on *cox11* growth**. (A) Time-resolved effect on growth of addition of the different amino acids. The size of the spots was quantified at each given timed and compared with the control by multiple two-way ANOVA; means comparison was done through Fisher LSD (least significant difference) test. For significant comparisons (p<0.05), the direction of growth is shown (Up, Down). Not significant comparisons are depicted as “n. s.”. (B) Area of the spots of *cox11* plants grown on $PpNO_3$ supplemented with amino acids (average ± SE, n>3). Only the amino acids that caused an improvement of growth in *cox11* are included.

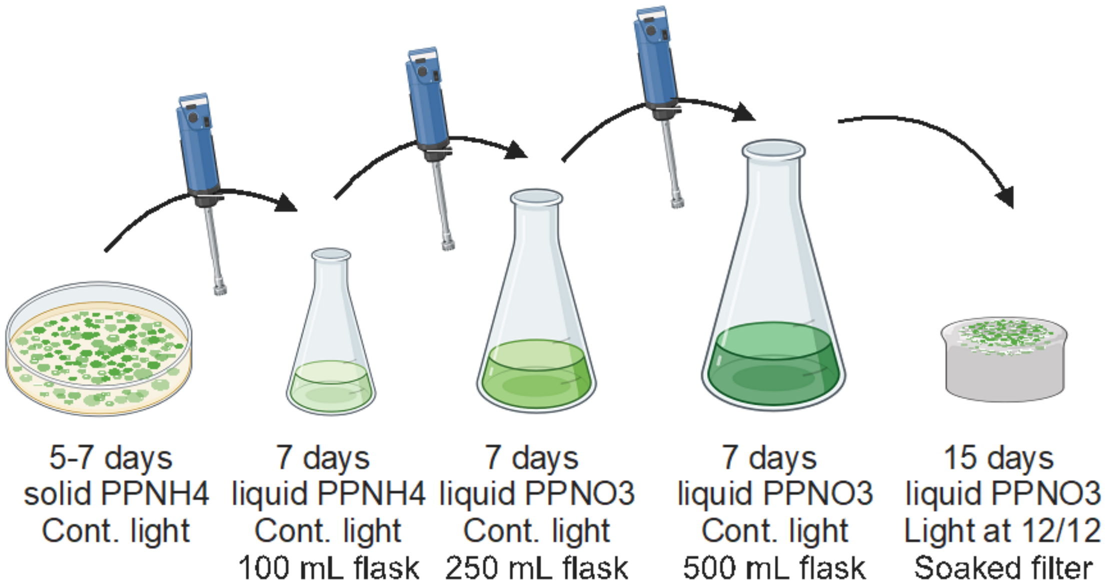

Supplementary Figure S12. Protocol for amplifying moss material for transcriptomics and metabolomics. The scheme presents how P. patens were amplified in liquid cultures before starting a hydroponic culture for metabolome analysis. Created with BioRender.com.

Supplementary Table S1. Relative expression of genes induced during UPR^{ER} in *P. patens*. Missing data (-) means that the gene was not detected as significantly expressed in any condition. (***) p<0.001; (**) p<0.01; (*) p<0.1; (n.s.) p>0.1 Source: Lloyd et al., 2018.

		ZT0	ZT0	ZT2	ZT2	ZT6	ZT6
ID in original ref	Gene ID	lfc	Signif.	lfc	Signif.	lfc	Signif.
Pp1s181_3V6	Pp3c10_17310	0.77	**	0.75	*	0.69	**
Pp1s566_63V6	Pp3c15_22880	0.66	*	0.39	n.s.	0.01	n.s.
Pp1s368_19V6	Pp3c20_14730	0.75	**	0.32	n.s.	-0.18	n.s.
Pp1s298_70V6	Pp3c4_21130	0.47	n.s.	0.51	n.s.	0.34	n.s.
Pp1s91_238V6	Pp3c12_8210	0.13	n.s.	0.25	n.s.	0.23	n.s.
Pp1s64_46V6	Pp3c5_8550	-0.50	n.s.	0.19	n.s.	0.23	n.s.
Pp1s213_66V6	Pp3c9_3330	0.14	n.s.	0.19	n.s.	0.26	n.s.
Pp1s34_189V6	Pp3c14_20430	0.13	n.s.	-0.15	n.s.	-0.03	n.s.
Pp1s241_31V6	Pp3c20_22800	-0.41	*	0.25	n.s.	0.46	*
Pp1s15_112V	Pp3c16_14150	-0.48	n.s.	0.31	n.s.	0.30	n.s.
Pp1s288_23V6	Pp3c3_25750	-	-	-	-	-	-
Pp1s34_31V6	Pp3c14_18900	-	-	-	-	-	-

Supplementary Table S2. Relative expression of antioxidant enzymes in *cox11*. Missing data (-) means that the gene was not detected as significantly expressed in any condition. (***) p<0.001; (**) p<0.01; (*) p<0.1; (n.s.) p>0.1. References: (a) Wu & Wang, 2019; (b) Higashi et al., 2013; (c) Y. J. Liu et al., 2013; (d) L. Xu et al., 2013.

				cox11 ZT0		cox11 ZT2		cox11 ZT6	
Symbol	Name	Ref	Gene ID	lfc	sig	lfc	sig	lfc	sig
PpAPX2 (PpAPX2.1)	Ascorbate peroxidase	a	Pp3c20_2050	0.51	*	0.27	n.s.	0.19	n.s.
PpAPX6-related	Ascorbate peroxidase	a	Pp3c17_7560	0.88	**	0.61	n.s.	0.73	*
PpAPX-S	Ascorbate peroxidase	a	Pp3c1_40650	0.13	n.s.	-0.60	*	-0.10	n.s.
PpAPX3	Ascorbate peroxidase	a	Pp3c1_26270	0.05	n.s.	-0.30	n.s.	0.11	n.s.
PpAPX2 (PpAPX2.2)	Ascorbate peroxidase	a	Pp3c20_2100	0.01	n.s.	0.28	n.s.	0.60	*
PpCSD2	CuZn-SOD - chloroplast (predicted)	b	Pp3c9_25690	0.35	n.s.	0.35	n.s.	0.51	n.s.
PpCSD1	CuZn-SOD - chloroplast (predicted)	b	Pp3c9_24840	0.02	n.s.	0.17	n.s.	0.90	*
PpCSD4	CuZn-SOD - cytosol (predicted)	b	Pp3c24_4100	0.60	*	0.39	n.s.	0.01	n.s.
PpCSD3	CuZn-SOD - cytosol (predicted)	b	Pp3c20_17920	0.37	n.s.	0.11	n.s.	-0.03	n.s.
PpFSD1	Fe-SOD - apoplast (predicted)	b	Pp3c7_19790	-0.26	n.s.	0.31	n.s.	1.37	***
PpFSD2	Fe-SOD - chloroplast (predicted)	b	Pp3c2_27440	1.16	***	1.07	***	1.43	***
PpFSD3	Fe-SOD - pseudogene	b	Pp3c17_14510	-2.06	n.s.	-2.03	n.s.	-2.97	*
PpGSTF7	Glutathione S-transferase	c	Pp3c7_13080	-0.34	n.s.	-0.83	n.s.	0.22	n.s.
PpGSTF2	Glutathione S-transferase	c	Pp3c3_31590	0.86	*	-0.05	n.s.	-0.25	n.s.
PpGSTF2	Glutathione S-transferase	c	Pp3c23_20550	-	-	-	-	-	-
PpGSTF5	Glutathione S-transferase	c	Pp3c17_6080	-	-	-	-	-	-
PpGSTF6	Glutathione S-transferase	c	Pp3c23_20550	-	-	-	-	-	-
PpGSTT3	Glutathione S-transferase	c	Pp3c7_26710	-	-	-	-	-	-
PpMSD	Mn-SOD - mitochondrion (predicted)	b	Pp3c19_10240	-0.02	n.s.	-0.06	n.s.	-0.12	n.s.
PpGR	Glutathione reductase	d	Pp3c4_17890	0.19	n.s.	0.27	n.s.	-0.05	n.s.
PpGR	Glutathione reductase	d	Pp3c5_16850	0.09	n.s.	-0.24	n.s.	0.13	n.s.

Supplementary Table S3. Primers used in this work. 3'→5' nucleotide sequences are given.

Primer name	Nucleotide sequence
Actin7 for	GCGAAGAGCGAGTATGACGAG
Actin7 rev	AGCCACGAATCTAACTTGTGAT
Cox11 p5	TGTTGTTCAAATGTCGTCAT
Cox11 p6	TGGACAAATTATTCCATGCT
Cox11-p1	CCTAGGTCGATGATTTGTGTTTTTGA
Cox11-p2	CTCGAGCCTATGGTTTGCAAGTAAGG
Cox11-p3	GTTAACACAAGCTCAGTACCCATCAC
Cox11-p4	TTAATTAAAAAAAGACGCAAACACAGAT
Cox11-p5	TGTTGTTCAAATGTCGTCAT
Cox11-p6	TGGACAAATTATTCCATGCT
Cox11-RTf	CATCCAGGGAATTGGTAGTA
Cox11-RTr	ATCTTCAAATCACGAAGGTG
HNZ Up Rev	TGCGCAACTGTTGGGAAG
35S terminator	CGCTGAAATCACCAGTCTCTCT

Supplementary Table S4. Antibodies used in this work. For each antibody, the target protein complex and subunit are indicated. For commercial antibodies, the item reference is included; for homemade antibodies, the original publication is cited.

Target complex	Target protein	Source/reference
Complex I	Nad9	Lamattina et al., 1993
Complex II	SDH 1-1	Peters et al., 2012
Complex III	MPP alpha	Peters et al., 2012
Complex V	Beta subunit	Peters et al., 2012
AOX	AOX	Agrisera AS04 054
PSII	Cp47	Storti et al., 2020
PSII	D2	Storti et al., 2020
PSI	PsaD	Agrisera AS09 461
Flv	FlvB	Gerotto et al., 2016
ATP synthase	γ ATPase	Agrisera AS08 312
PSII	PsbS	Storti et al., 2020
PSI	PsaA	Agrisera AS06 172
Cyt b6/f-complex	Cyt f	Agrisera AS06 119

Supplementary Table S5. Available information on ascorbate peroxidase encoding genes in *P. patens*. Information on gene name and predicted localization was retrieved from literature. Name in the most recent gene model version is given in the first column. Localization as described in each of the references are included: Cyt, cytosol; Chlo, chloroplast; Mito, mitochondrion; Perox, peroxisome.

Reference	Ozyigit et al., 2016		Maruta et al., 2016		Wu & Wang, 2019	
Gene ID	Name	Localization	Name	Localization	Name	Localitzation
Pp3c1_26270	Phpat.001G104200	Chlo/Cyt	APX3	Perox	PpAPX3	Perox
Pp3c1_40650	Phpat.001G162800	Chlo	APX4	Chloro	PpAPX-S	Mito/Chloro
Pp3c17_7560	Phpat.017G025400	Chloro	-	-	PpAPX6-related	Chloro
Pp3c20_2050	Phpat.020G011100	Cyto	APX1	Cyto	PpAPX2.1	Cyto
Pp3c20_2100	-	-	APX2	Cyto	PpAPX2.2	Cyto
